# Structural basis of antigenic drift in FMDV O/Cathay topotype variants revealed by porcine-derived single B-cell neutralizing antibodies

**DOI:** 10.1371/journal.ppat.1014650

**Published:** 2026-10-15

**Authors:** Fengjuan Li, Shanquan Wu, Shulun Huang, Lv Lv, Yimei Cao, Huifang Bao, Pu Sun, Xingwen Bai, Jiaxin Yang, Jingjing Zha, Qiongqiong Zhao, Hehe Zhang, Dong Li, Jijun He, Zaixin Liu, Pinghua Li, Zengjun Lu, Dongsheng Lei, Kun Li

**Affiliations:** 1 State Key Laboratory of Animal Disease Control and Prevention, College of Veterinary Medicine, Lanzhou University, Lanzhou Veterinary Research Institute, Chinese Academy of Agricultural Sciences, Lanzhou, China; 2 Gansu Province Research Center for Basic Disciplines of Pathogen Biology, Lanzhou, China; 3 Key Laboratory of Magnetism and Magnetic Functional Materials, School of Physical Science and Technology, Electron Microscopy Centre of Lanzhou University, Lanzhou University, Lanzhou, China; 4 Jiangsu Key Laboratory of Zoonosis, Yangzhou University, Yangzhou, China; CSIC: Consejo Superior de Investigaciones Cientificas, SPAIN

## Abstract

The porcinophilic O/Cathay topotype variants of foot-and-mouth disease virus (FMDV) continuously accumulated mutations over time and produced antigenic drift, leading to diminished serum neutralization and compromised vaccine efficacy. However, the structural basis underlying this divergence remains poorly defined. Herein, we observed that the newly emerging O/Cathay variants in China exhibited marked neutralization escape from porcine immune sera. Furthermore, using a panel of seven porcine-derived neutralizing monoclonal antibodies (pnAbs), we mapped two antigenic regions with crucial determinants responsible for neutralization escape in recent O/Cathay variants. The charge change at +2 residue (Q → H/R) downstream of the conserved RGD receptor-binding motif on VP1, which modulates sensitivity to multiple pnAbs (pO18-34, pO18-40 and pO18-43) and vaccine-induced sera. Cryo-electron microscopy (cryo-EM) structures of pO18-29 in complex with the ancient O/HN/CHA/93 strain and the recent variant O/18074 revealed a novel VP3-centered antigenic structure and showed that mutations at positions 73 (V → K) and 134 (K → T) on VP3 mediated neutralization escape in O/Cathay variants via disruption of hydrophobic interaction as well as the hydrogen bonds and salt bridge networks. Moreover, residues 73 and 134 synergistically regulate pnAbs (pO18-2, pO18-18, pO18-28 and pO18-29) recognition. Together, our findings elucidate structural basis of neutralizing evasion in O/Cathay FMDV and provide a molecular framework for the rational design of next-generation broad-spectrum FMDV vaccine against emerging O/Cathay variants.

## Author summary

Foot-and-mouth disease virus (FMDV) remains a major threat to livestock production worldwide, and the emergence of new variants can undermine the effectiveness of existing vaccines. In this study, we examined newly emerging O/Cathay FMDV variants circulating in China and found that they can evade neutralization by immune sera from vaccinated pigs. By analyzing a panel of pig-derived neutralizing monoclonal antibodies, we identified two antigenic regions that play distinct roles in mediating this neutralization escape. A charge-changing mutation near the conserved RGD receptor-binding site on the viral capsid protein VP1 reduces antibody recognition. Cryo-electron microscopy structures further revealed a previously unrecognized antigenic surface on the VP3 protein. We show that mutations at positions 73 and 134 on VP3 disrupt critical molecular interactions, including hydrophobic contacts, hydrogen bonds, and salt bridges, thereby enabling the virus to escape multiple antibodies binding. Notably, these two residues on VP3 act cooperatively to regulate antibody recognition. Together, our findings provide structural insights into how emerging FMDV variants evade immune responses and highlight specific targets for the design of broadly protective vaccines against evolving O/Cathay strains.

## Introduction

Foot-and-mouth disease (FMD) is a highly contagious viral disease affecting cloven-hoofed animals, including pigs, cattle, and sheep, and poses a major threat to animal health and international trade [1]. The causative agent, foot-and-mouth disease virus (FMDV), comprises seven immunologically distinct serotypes, with serotype O being the most prevalent and antigenically diverse [2,3]. In Asia, recent epidemics have primarily been caused by three topotypes of serotype O, including Middle East-South Asia (ME-SA), Southeast Asia (SEA), and Cathay (CHY) [4,5]. Notably, Cathay topotype viruses have shown an accelerated evolutionary rate and increased genetic diversity in recent years, raising concerns about their ongoing antigenic variation [6].

The Cathay topotype represents a distinct lineage of serotype O FMDV with unique biological and epidemiological features. Strains of Cathay topotype display a pronounced porcinophilic phenotype and harbor characteristic deletions in both the 5′ untranslated region (UTR) and the non-structural protein 3A gene [7,8]. Since its initial emergence in Hong Kong in the 1970s [9], the O/Cathay lineage has repeatedly caused outbreaks in swine populations, including the large-scale epidemic in Taiwan in 1997 [10,11]. Over the decades, Cathay strains have continued to circulate across East and Southeast Asia, and diversified into multiple independently evolving sub-lineages [12–14]. As a consequence of this sustained circulation and diversification, recent O/Cathay viruses have exhibited pronounced antigenic drift, leading to a marked reduction in the effectiveness of conventional vaccines [6]. Vaccination remains the cornerstone of FMD prevention and control; however, the antigenic variation creates a major problem in controlling FMD outbreaks by using conventional inactivated virus vaccines [15]. Although current serotype O inactivated vaccines effectively protect against ME-SA and SEA viruses, newly emerged O/Cathay variants exhibit markedly reduced antigenic match and frequently escape vaccine-induced neutralization [16].

Antigenic drift in FMDV follows several key principles. Minimal structural changes, most commonly single amino acid substitutions in side chains that alter size or charge, can disrupt antibody binding without altering backbone conformation or compromising capsid integrity and function [17]. These substitutions are spatially clustered and predominantly occur at five major surface-exposed antigenic sites (sites 1–5) located on the capsid proteins VP1, VP2, and VP3 [18,19]. Pronounced antigenic drift often involves co-variation, in which coordinated substitutions across multiple antigenic sites, particularly sites 1, 2, and 5, collectively remodel antigenic epitopes on FMDV capsids [20]. Consistent with these principles, antigenic drift in O/Cathay viruses has been associated with substitutions at multiple antigenic residues. The substitutions in VP1 (positions 141, 147, and 149) and VP2 (position 130) contribute to immune escape [5]. A VP2 S72N substitution was also identified as a key antigenic determinant in O/Penghu/2012 using a mouse-derived monoclonal antibody [21]. However, most existing analyses rely on murine antibodies and lack structural resolution, limiting mechanistic insight into antibody recognition and escape in the natural porcine host.

In this study, we employed a panel of seven porcine-derived neutralizing monoclonal antibodies (pnAbs) to characterize the antigenic architecture of recent O/Cathay strains. We show that Cathay variants circulating between 2016 and 2022 exhibit substantial escape from neutralization by both porcine immune sera and monoclonal antibodies. We identify the key antigenic determinant of neutralization escape approaching the receptor binding region on VP1. Cryo-EM complex structures uncovered a previously uncharacterized VP3-centered conformational epitope and demonstrated a synergistic escape involving VP3 residues 73 and 134. These findings provide insights into the molecular mechanisms driving antigenic drift in O/Cathay variants and offer a structural framework for rational vaccine updates to improve protection in swine populations in Asia.

## Results

### The newly emerging O/Cathay variants exhibited marked neutralization escape from porcine immune sera

To evaluate the antigenic variation of the newly emerged O/Cathay strains (O/SCGH/CHA/2016, O/18074, O/GD/2021 and O/GX/2022) isolated between 2016 and 2022, cross-neutralization assays were performed using porcine sera from four distinct immunized groups. Each group consisted of ten pigs that had received two doses of inactivated vaccines containing 146S antigens derived from the O/HN/CHA/93 (Cathay), O/XJ/CHA/2017 (O/Ind/2001 lineage), O/Tibet/99 (O/PanAsia lineage), or O/GSLX/2010 (O/Mya98 lineage), respectively. Compared with the ancient O/HN/CHA/93 strain, all four O/Cathay variants exhibited substantial escape from serum neutralization across all immunized groups (Fig 1). In the O/HN/CHA/93-immunized group, the mean neutralizing titers against O/SCGH/CHA/2016, O/18074, O/GD/2021 and O/GX/2022 strains were decreased by 11.8-, 14.0-, 15.2- and 24.5-fold, respectively, relative to those against O/HN/CHA/93 strain (Fig 1A). A similar pattern was also observed in the O/Ind/2001-immunized group, with the lowest titers observed against O/GX/2022 strain, followed by O/GD/2021, O/18074 and O/SCGH/CHA/2016 strains (Fig 1B). In contrast, sera from pigs immunized with the O/Tibet/99 or O/Mya98 showed lower neutralization against the O/SCGH/CHA/2016 than against O/18074, whereas neutralization of O/GD/2021 and O/GX/2022 remained consistently low (Fig 1C-D). The radar plot further highlights a pronounced decline in neutralizing titers against the four recent O/Cathay variants compared with those against O/HN/CHA/93 (Fig 1E).

**Fig 1:**
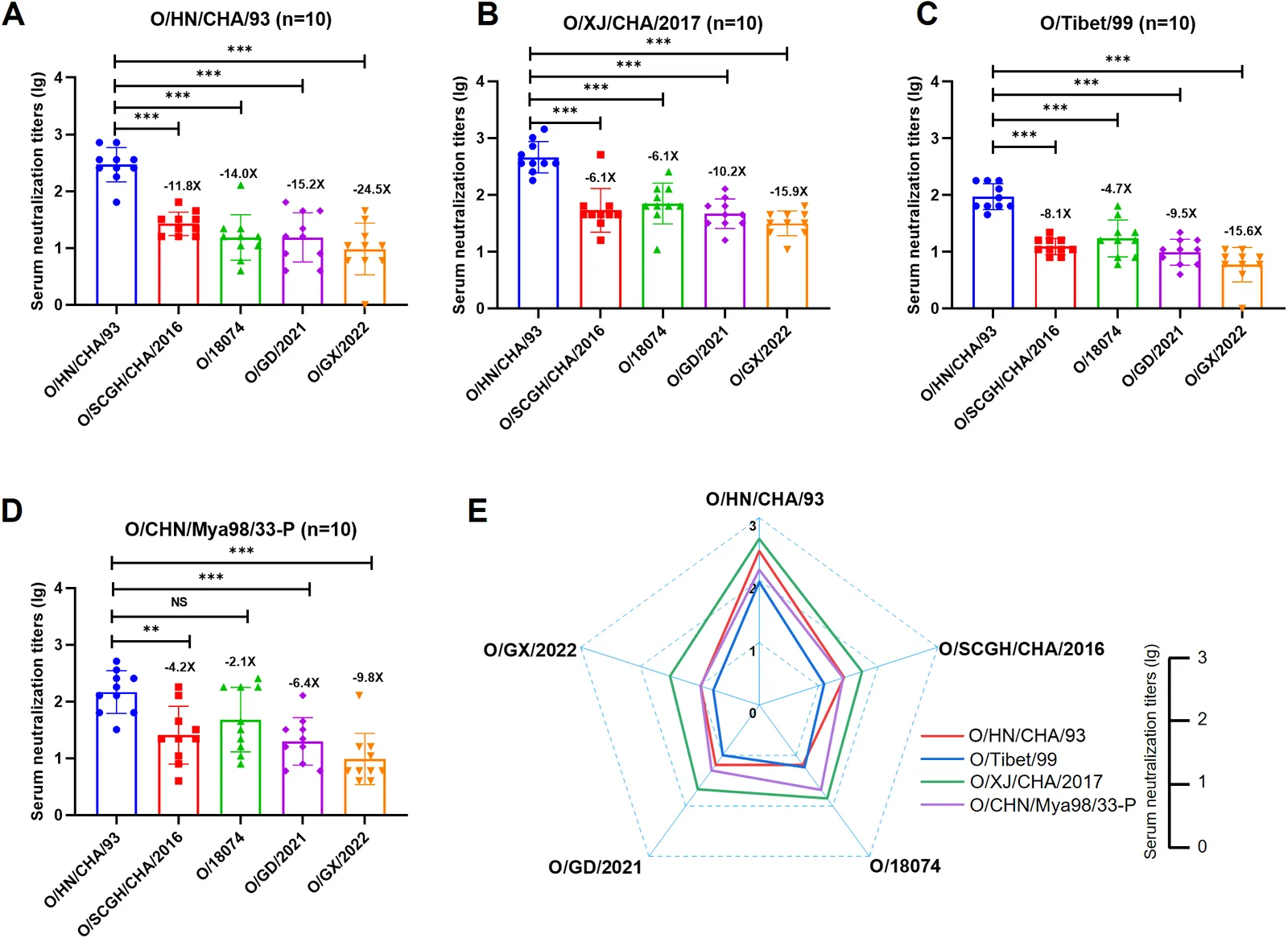
Neutralization of O/Cathay variants by porcine sera. **(A-D)** The neutralization titers of ancient O/HN/CHA/93 strain and representative O/Cathay variants were determined by microneutralization assay using porcine sera collected from four different immunization groups vaccinated with four different serotype O virus antigens. The O virus antigens are O/HN/CHA/93 (Cathay), O/XJ/CHA/2017 (O/Ind/2001 lineage), O/Tibet/99 (O/PanAsia lineage), or O/GSLX/2010 (O/Mya98 lineage). The inactivated vaccine strains and the sample size (n) for each group are indicated in the panel titles. Fold changes in geometric mean neutralization titers against each variant relative to the O/HN/CHA/93 strain are indicated by symbols. Statistical differences between O/HN/CHA/93 and each O/Cathay variant were analyzed with one-way ANOVA followed by Dunnett's multiple-comparison test in GraphPad Prism 7.2. ***P < 0.001; **P < 0.01; *P < 0.05. Experiments were performed independently in triplicate. **(E)** Radar plot showing the serum neutralization profiles for four immunized groups. Groups of three-month old healthy pigs (n = 10) were immunized with the indicated vaccine formulations.

To further assess whether these findings extend to other topotypes of serotype O, we evaluated cross-neutralization of the recently emerged ME-SA (O/GZ1/2025) and SEA (O/HNNY/2022) strains using the same panel of porcine sera. Notably, the neutralization profiles of these two strains differed markedly from those of O/Cathay variants. O/HNNY/2022 escaped neutralization by O/HN/CHA/93 (4.7-fold), O/Tibet/99 (2.3-fold), and O/Ind/2001 (2.0-fold) sera (Fig A panels A-C in S1 Appendix), but showed enhanced neutralization (0.6-fold) by O/Mya98 sera (Fig A panel D in S1 Appendix). In contrast, O/GZ1/2025 exhibited marginal escape from O/HN/CHA/93 (2.6-fold) and O/Mya98 (1.4-fold) sera, while displaying enhanced neutralization by O/Tibet/99 (0.9-fold) and O/Ind/2001 (0.8-fold) sera (Fig A in S1 Appendix). Among the four vaccine groups, sera from pigs immunized with the O/Ind/2001 exhibited consistently higher neutralizing activity against all tested strains, indicating broader cross-reactivity.

Notably, phylogenetic analysis of the P1 region showed that O/Cathay variants from 2016–2022 formed a distinct clade, with O/GD/2021 and O/GX/2022 occupying the most divergent branches (Fig B panel A in S1 Appendix). This correlates directly with their rank order of neutralization resistance observed in Fig 1 (O/GX/2022 > O/GD/2021 > O/18074 > O/SCGH/CHA/2016), providing evidence that genetic drift in P1 is associated with progressive antigenic change within the Cathay topotype. Collectively, these results demonstrate that O/GX/2022 exhibits the greatest resistance to porcine serum neutralization, followed by other O/Cathay variants, highlighting increasing antigenic divergence within Cathay topotype.

### Reduced binding and neutralization of O/Cathay variants were further confirmed by porcine monoclonal antibodies

We selected 55 mAb clones from the previously established O/18074-specific porcine BCR repertoire (Fig 2A) [12], derived from a pig primed and boosted with O/HN/CHA/93 and then immunized with O/18074, O/Tibet/99, A/WH/CHA/09, and A/AF72. These clones were chosen based on high clonal frequency and somatic hypermutation (SHM) profiles and were derived from the unique 2014 O-specific clonotypes after excluding clonotypes shared with the serotype A repertoire. Seven representative porcine mAbs (designated as pO18-2, pO18-18, pO18-28, pO18-29, pO18-30, pO18-34 and pO18-43) were expressed for further analysis. The binding reactivity of these mAbs to the ancient O/Cathay strain O/HN/CHA/93 and the recent variant O/18074 was evaluated using indirect immunofluorescence assay (IFA) and biolayer interferometry (BLI). All mAbs recognized O/HN/CHA/93 in IFA, although with varying intensities (Fig 2B). PO18-2 and pO18-43 showed weak fluorescence signals, whereas the remaining mAbs displayed strong reactivity. All mAbs exhibited only weak binding to O/18074 in IFA (Fig C in S1 Appendix). BLI kinetic analyses revealed that all mAbs bound O/HN/CHA/93 with greater affinity than the recent variant O/18074 (Fig 2C-D), with slower dissociation rates observed for O/HN/CHA/93. Among them, pO18-28 showed the largest difference in affinity, with a dissociation constant (KD) of 0.246 nM for O/HN/CHA/93, and 11 nM for O/18074. PO18-29 exhibited the greatest difference in dissociation rate, with k_off values of 1.90 × 10^−4^ s^−1^ for O/HN/CHA/93, and 5.62 × 10^−3^ s^−4^ for O/18074. pO18-43 showed a smaller difference than other mAbs, with an approximately threefold change in k_off value. Overall, all mAbs showed lower binding affinity to O/18074 than to O/HN/CHA/93 (Fig 2E). The higher affinity of O/18074-derived mAbs for the ancient strain O/HN/CHA/93 than for the homologous variant is consistent with original antigenic sin [22,23], a phenomenon in which initial exposure to an ancestral strain shapes subsequent antibody responses toward cross-reactive epitopes.

**Fig 2:**
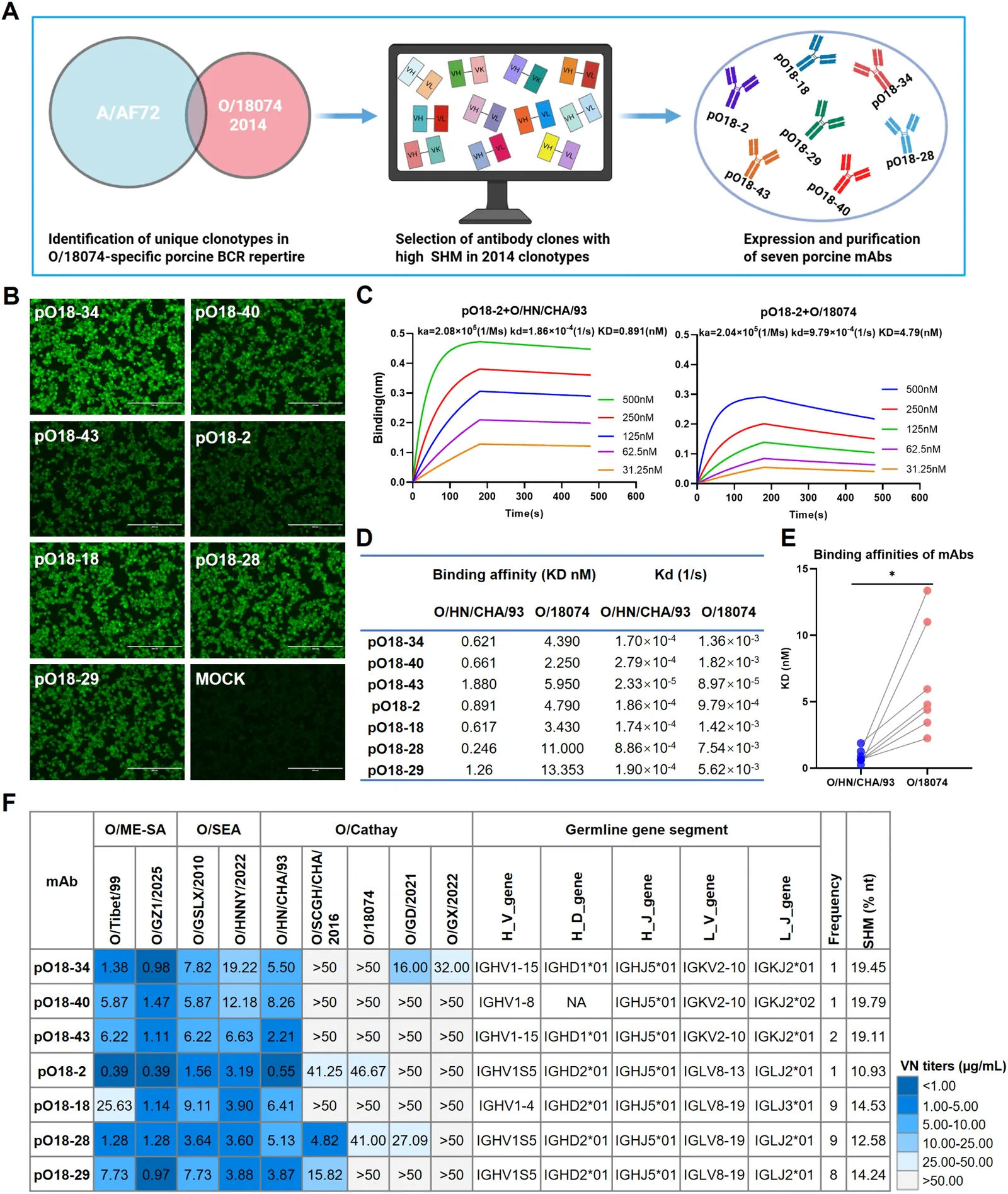
Functional characterization of porcine-derived monoclonal antibodies against FMDV. **(A)** Schematic diagram of selection and preparation of porcine mAbs from O/18074 strain-specific BCR repertoire. Seven porcine monoclonal antibodies (mAbs) specific to FMDV serotypes O, were selected and expressed in 293-F cells. Created in BioRender. Chen, Y. (2026) https://BioRender.com/6eqcupv. **(B)** Reactivity of porcine mAbs against FMDV serotype O was assessed by indirect immunofluorescence assay (IFA). BHK-21 cells infected with the O/HN/CHA/93 strain were incubated with porcine mAbs (5 μg/mL), followed by detection with FITC-conjugated rabbit anti-pig IgG. Images were acquired using a fluorescence imaging system. Experiments were performed independently in triplicate. **(C, D)** Binding affinities of porcine mAbs to inactivated 146S antigens of ancient O/HN/CHA/93 strain and representative O/Cathay variant O/18074 were measured by BLI. Representative binding plot were shown for pO18-2 **(C)**, and the kinetic parameter (k_d) and equilibrium dissociation constants (K_D) for all mAbs are summarized **(D)**. **(E)** Differences in binding affinities of the seven mAbs between O/HN/CHA/93 and O/18074 were statistically analyzed. Statistical differences between O/HN/CHA/93 and O/18074 were assessed using a paired t-test (parametric) in GraphPad Prism 7.2. ***P < 0.001; **P < 0.01; *P < 0.05. **(F)** Neutralizing activities of porcine mAbs against representative serotype O strains were evaluated by microneutralization assay and are expressed as virus neutralization (VN) titers (μg/mL), shown with a color scale. The immunoglobulin germline gene usage (VH, DH, JH, Vλ, Jλ) was identified and characterized. Frequency indicated the frequency of clonotypes for each mAb that appeared in O/18074-specific repertoire. SHM indicated frequency of replacement and silent mutations in full variable region sequence of heavy chain (VH), which was calculated by SHazaM using the program Immcantation.

We then assessed neutralization breadth and potency of these mAbs using virus neutralization assay (VNT) against the representative epidemic strains of FMDV serotypes O circulating in China, including O/Tibet/99 and O/GZ1/2025 (ME-SA topotype), O/GSLX/2010 and O/HNNY/2022 (SEA topotype), and five strains (O/HN/CHA/93, O/SCGH/CHA/2016, O/18074, O/GD/2021 and O/GX/2022) in Cathay topotype as well as serotype A strains (A/AF72, A/WH/CHA/09, and A/GDMM/2013). All seven mAbs exhibited broad neutralizing activity against O/Tibet/99, O/GZ1/2025, O/GSLX/2010, O/HNNY/2022 and O/HN/CHA/93 strains in the VNT. In contrast, neutralizing activity of these mAbs was markedly reduced against Cathay variants isolated after 2016, with several mAbs completely losing neutralizing activity against O/GD/2021 and O/GX/2022. None of these mAbs could neutralize the tested FMDV serotype A strains.

Among these mAbs, pO18-2 demonstrated potent neutralizing activity against O/HN/CHA/93 (<1 μg/mL), but exhibited markedly weaker activity against O/SCGH/CHA/2016 (41.25 μg/mL) and O/18074 (46.67 μg/mL), and completely lost activity against O/GD/2021 and O/GX/2022 (>50 μg/mL) (Fig 2F). The broad neutralization profiles of these mAbs further indicate that the recently emerged ME-SA (O/GZ1/2025) and SEA (O/HNNY/2022) strains have not undergone significant antigenic variation. The reduced neutralization correlated with decreased binding affinity for the variants, underscoring the substantial antigenic variation that has emerged among O/Cathay strains after 2016.

### The charge change at RGD + 2 position (Q → H) significantly affected antigenic variation and occurred in emerging variants of O/Cathay topotype

To illustrate key determinants of antigenic variation in O/Cathay strains emerging after 2016, we generated neutralization escape mutants against the seven pnAbs using the O/HN/CHA/93 strain as the parental virus. As summarized in Table 1, escape mutations were mapped to VP1 for three pnAbs, whereas mutations selected by the remaining four pnAbs localized in VP3. Escape mutants selected by pO18-34, pO18-40, and pO18-43 harbored substitutions within the VP1 G-H loop. Notably, these substitutions clustered downstream of the RGD motif, which constitutes the integrin receptor-binding site of FMDV. A total of 24 mutants contained substitutions at VP1 position 149 (RGD + 2). Most substitutions (14/24) involved a change from glutamine (Q) to arginine (R), with additional substitutions to lysine (K), histidine(H), or leucine (L) (Table 1). Sanger sequencing of the escape mutants confirmed the presence of these mutations in the viral stocks (Figs D-F in S1 Appendix). We further performed amplicon sequencing to quantify the mutation frequency in the viral stocks. For three representative escape mutants (one each for pO18-34, pO18-40, and pO18-43), all three exhibited mutations at P1 position 2015 (corresponding to VP1 residue 149), with a mutation frequency exceeding 90% (Fig G in S1 Appendix). For the pO18-43 escape mutant, additional mutations were detected at P1 positions 489, 819, and 135, which were synonymous and present at frequencies below 1%. Interestingly, these substitutions predominantly altered amino acid charge or polarity, and the majority represented a shift from neutral to basic residues. Sequence alignment of FMDV serotype O viruses revealed a consistent residue difference at VP1 position 149 between ancient and recent strains. The ancient O/HN/CHA/93 strain carries Q149, whereas O/Cathay variants after 2016 predominantly carry H149 (Fig 3A).

**Table 1:** Porcine neutralizing mAb escape mutants.

mAb	Parent virus	Residue change(s)	^$^Frequency of mutants	^#^Neutralization Concentration (µg/mL)
pO18-34	O/HN/CHA/93	VP1 Q149R	5/8	>400
		VP1 Q149K	1/8	>400
		VP1 R140H, Q149L	1/8	>400
		VP1 Q149L	1/8	>400
pO18-40	O/HN/CHA/93	VP1 Q149R	4/8	>400
		VP1 Q149L	1/8	>400
		VP1 Q149H	1/8	>400
		VP1 Q149K	1/8	>400
		VP1 L112I, Q149K	1/8	>400
pO18-43	O/HN/CHA/93	VP1 Q149R	5/8	>400
		VP1 Q149L	2/8	>400
		VP1 Q149K	1/8	>400
pO18-18	O/HN/CHA/93	VP3 D71G, K134E	2/8	>400
		VP3 D71N, K134E	2/8	>400
		VP3 D70L, K134E	2/8	>400
		VP3 K134E	1/8	>400
		VP3 D71H	1/8	>400
pO18-2	O/HN/CHA/93	VP3 Q76R	3/8	>400
		VP3 Q76R, T99A, K134E	1/8	>400
		VP3 Q76R; VP1 A199D	1/8	>400
		VP3 S70P, E131G	1/8	>400
		VP3 S70P, K134E	1/8	>400
		VP3 S70P, Q76R	1/8	>400
pO18-28	O/HN/CHA/93	VP3 S70L, Q76R	1/8	>400
		VP3 S70P, Q76R	1/8	>400
		VP3 E58K, Q76R	1/8	>400
		VP3 V61I, VP1 G63E, D85A, A127S	1/8	>400
		VP2 K88R; VP3 Q76R	1/8	>400
		VP3 V61I; VP1 H108R	1/8	>400
		VP1 H108R	2/8	>400
pO18-29	O/HN/CHA/93	VP3 S70L, Q76R	4/8	>400
		VP3 S70P, Q76R	3/8	>400
		VP3 S70A, Q76R	1/8	>400

$ Frequencies of the mutants are the number of mutants with the mutation at the indicated residue/total number of mutants obtained.

# Neutralization concentration was determined as the lowest antibody concentration that protected cells from CPE.

**Fig 3:**
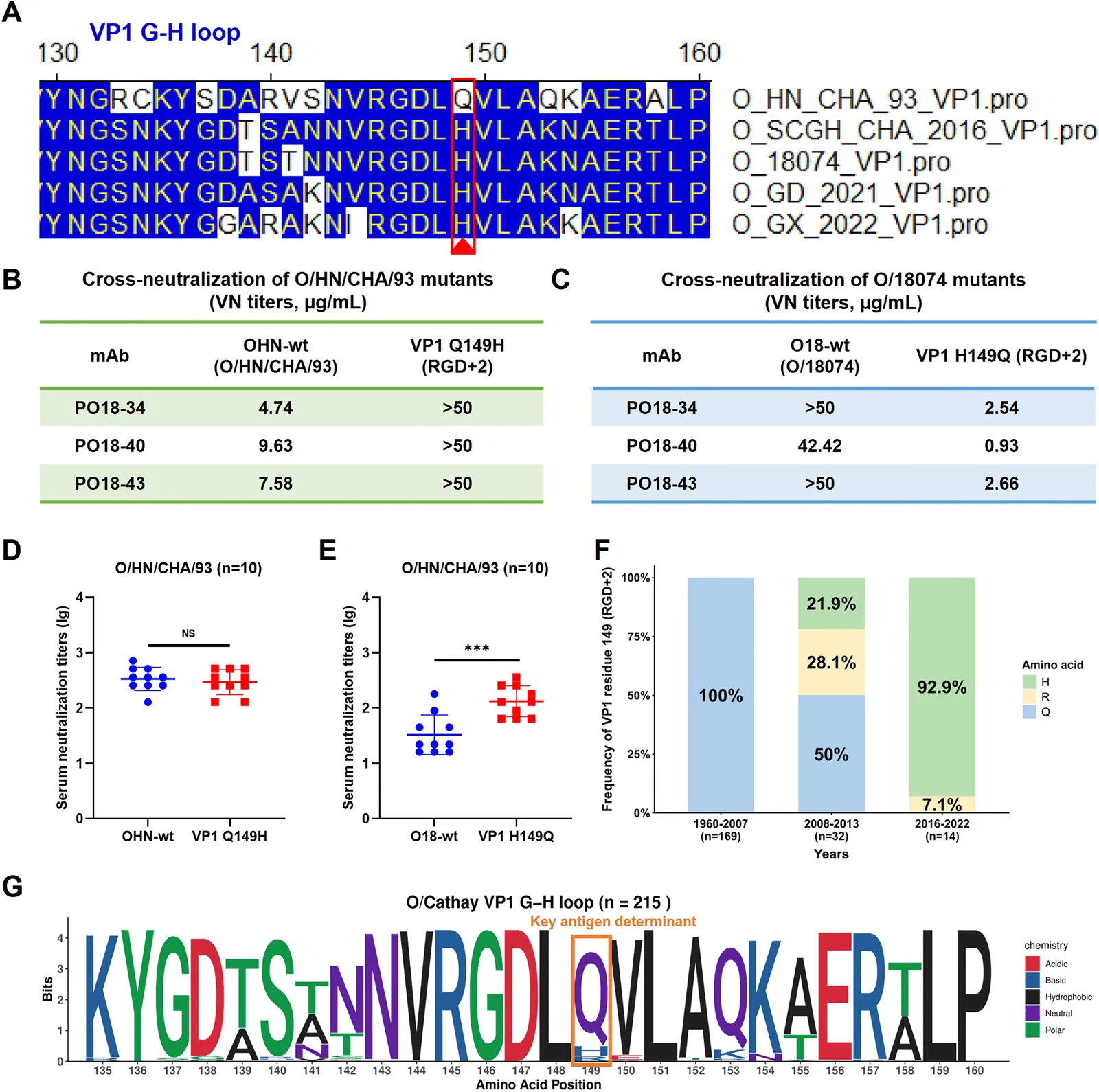
Identification of key variant residues in O/Cathay strains using porcine neutralizing antibodies. **(A)** Sequence alignment of the VP1 G-H loop from variants and ancient strain. Residue 149 (RGD + 2) is highlighted within a red box. **(B, C)** Identification of key antigenic determinants recognized by three pnAbs using rescued single-mutant FMDV strains. Neutralizing activities of pnAbs against O/HN/CHA/93 and its mutant **(B)**, as well as the O/18074 and corresponding mutant **(C)** were evaluated by microneutralization assay. **(D, E)** The neutralization titers of O/HN/CHA/93 **(D)** or O/18074 **(E)** and their mutants were determined by microneutralization assay using sera from O/HN/CHA/93-immunized pigs. The inactivated vaccine strain and the sample size (n) are indicated in the panel titles. Statistical differences were analyzed using an unpaired *t* test with the Holm-Sidak method (α = 0.05) in GraphPad Prism 7.2. ***P < 0.001; **P < 0.01; *P < 0.05; NS, not significant. Experiments were performed independently in triplicate. **(F)** Temporal distribution of VP1 residue 149 (RGD + 2) (percentage frequency) of O/Cathay strains. **(G)** The sequence logo of VP1 G-H loop of the O/Cathay strains. (n = 215 full-length VP1 sequences) retrieved from the National Center for Biotechnology Information (NCBI) database as of January 14, 2026.

To further validate the functional relevance of this residue, recombinant viruses bearing VP1 residue 149 substitutions were rescued and confirmed by Sanger sequencing and plaque assays (Fig H in S1 Appendix), and subsequently assessed for their sensitivity to neutralization by three pnAbs individually. In the O/HN/CHA/93 background, introduction of Q149H substitution completely abolished neutralization by pO18-34, pO18-40, and pO18-43 (Fig 3B). Accordingly, the reciprocal H149Q substitution in O/18074 background markedly restored neutralization by all these pnAbs (Fig 3C). Next, we assessed the impact of this substitution on polyclonal antibody responses *in vivo*, and sera from O/HN/CHA/93-immunized pigs were examined in cross-neutralization assays against the recombinant viruses of O/HN/CHA/93 with Q149H substitution (_OHN-VP1_Q149H) and O/18074 with H149Q substitution (_O18-VP1_H149Q) mutant viruses. No obvious difference in neutralizing antibody titers was observed between the porcine sera against the WT strain and the _OHN-VP1_Q149H mutant (Fig 3D). However, neutralizing antibody titers to _O18-VP1_H149Q mutant virus were significantly higher than those against parental virus O/18074 (Fig 3E). These results consolidate that VP1 residue 149 (RGD + 2) plays a pivotal role in modulating monoclonal antibody recognition and exerts a substantial influence on polyclonal serum neutralization.

Consistent with these functional observations, global sequence analyses revealed a clear temporal shift at VP1 residue 149 (RGD + 2) in O/Cathay viruses, evolving with time from glutamine (Q) in ancient strains to arginine (R), and predominantly histidine (H), in more recent variants (Fig 3F-G). Collectively, these findings establish VP1 residue 149 (RGD + 2) as a key determinant of antibody recognition and a major antigenic site in recent O/Cathay strains.

### Cryo-EM complex structures of virus-antibody revealed novel antigenic regions on VP3, with similar antigen footprints, but different numbers of hydrogen bonds and salt bridge networks between the ancient strain and variant in O/Cathay topotype

Escape mutants selected by pO18-2, pO18-18, pO18-28, and pO18-29 all harbored substitutions in VP3 (Table 1). None of these four pnAbs bound to denatured viral antigens by WB, indicating that they recognize conformational epitopes on surface of viral capsid. To dissect the structural basis of neutralization escape in emerging O/Cathay variants, we selected pO18‑29 for cryo‑EM analysis, as it exhibited potent neutralization against the ancestral O/HN/CHA/93 strain but not against the recent variants (2016–2022). We resolved cryo-EM structures of the pO18-29 single-chain variable fragment (scFv) in complex with capsids from the ancient strain O/HN/CHA/93 (FMDV-OHN–pO18-29) and the recent variant O/18074 (FMDV-O18–pO18-29), respectively. In both reconstructions, 60 copies of the scFv were symmetrically arranged around the threefold axes of the viral capsid (Fig 4A-B). The final reconstructions reached resolutions of 2.24 Å for FMDV-OHN–pO18-29 and 2.34 Å for FMDV-O18–pO18-29, as estimated by the 0.143 FSC criterion (Fig I in S1 Appendix), enabling reliable atomic modeling of the FMDV capsid proteins and the variable loops of the scFv antibody. Structural superposition of the two complexes yielded an RMSD of 1.432 Å (Fig 4C), indicating that antibody engagement adopts a nearly identical global architecture in both strains. The two complexes exhibited highly similar overall antigenic footprints (Fig 4D-E). The buried surface areas were comparable (1089.63 Å² for O/HN/CHA/93 versus 1005.04 Å² for O/18074) (Tables A and B in S1 Appendix).

**Fig 4:**
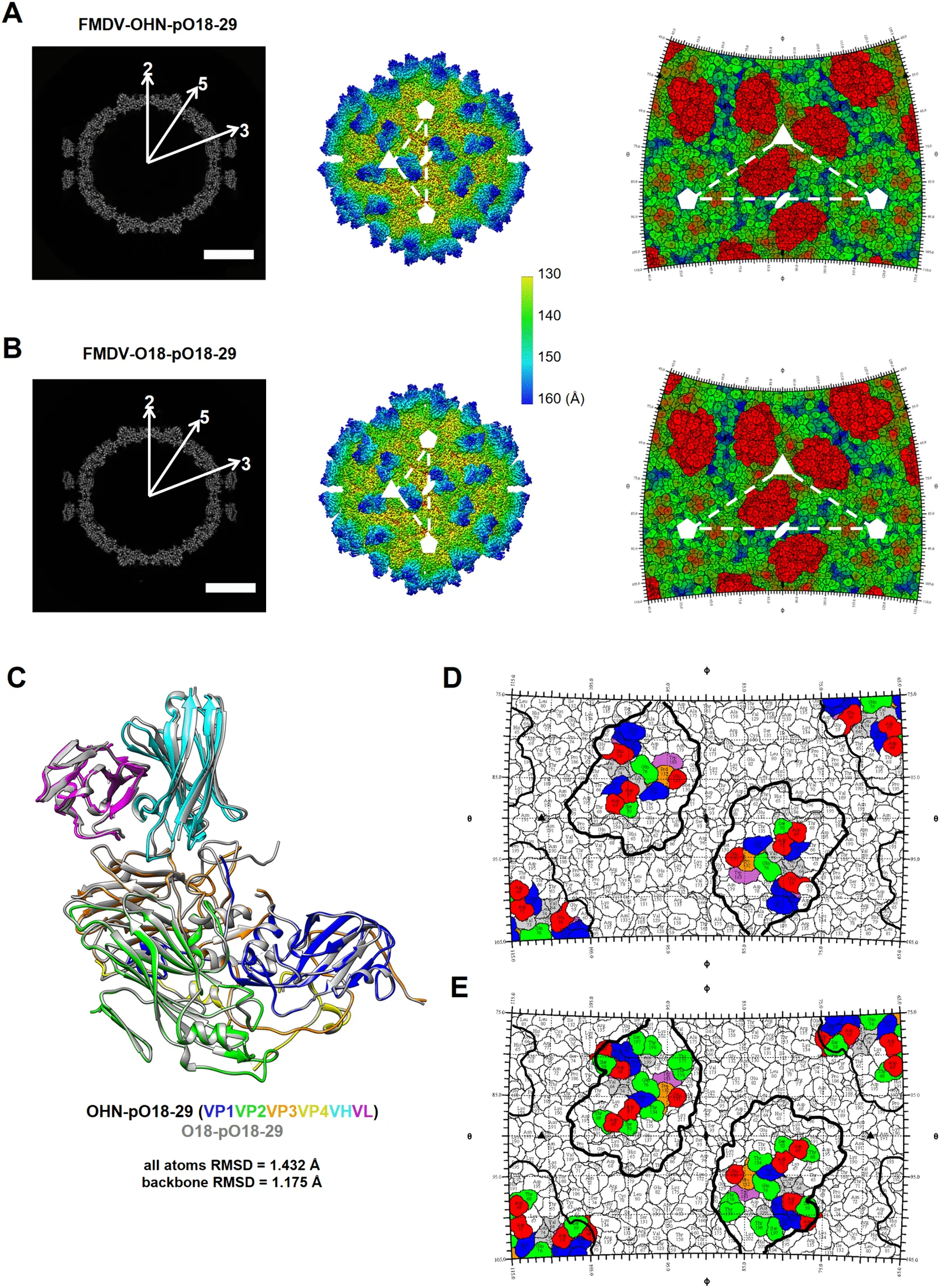
Cryo-EM analysis of the FMDV-OHN-pO18-29 and FMDV-O18-pO18-29 complexes. **(A, B)** The central cross sections, rendered images, and antibody footprints of the FMDV-OHN-pO18-29 complex **(A)** and the FMDV-O18-pO18-29 complex **(B)**. The central cross sections of the cryo-EM maps are shown with icosahedral 2-, 3-, and 5-fold axes (Scale bar, 10 nm). In the rendered images, depth cueing with color is used to indicate the radius (< 100 Å, blue; 120–160 Å, from cyan to yellow; > 180 Å, red). The icosahedral 5- and 3-fold axes are represented by pentagons and triangles, respectively. Footprints of pO18-29 on the FMDV surface were produced using RIVEM [54]. **(C)** Structural superposition of the FMDV-OHN–pO18-29 and FMDV-O18–pO18-29 complexes, aligned based on the FMDV capsid. The heavy and light chains of pO18-29 are colored cyan and magenta, respectively. Capsid proteins VP1, VP2, VP3, and VP4 are colored blue, green, red, and yellow, respectively. The all-atom root-mean-square deviation (RMSD) of pO18-29 is 1.432 Å, and the backbone RMSD is 1.175 Å. **(D, E)** Footprint maps of residues within 4 Å of the antibody with FMDV-OHN-pO18-29 **(D) or** FMDV-O18-pO18-29 **(E)** within 4 Å. Residues are colored according to physicochemical properties: H, K, and R, positive (blue); D and E, negative, hydrophilic (red); S, T, N, and Q, neutral, hydrophilic (green); A, V, L, I, and M, aliphatic, hydrophobic (gray); F, Y, and W, aromatic (purple); P and G, proline and glycine (orange). The antibody interface is outlined by a black contour line.

Despite this conserved global footprint, the underlying intermolecular interaction networks differed markedly in composition and organization. In the FMDV-OHN–pO18-29 complex, pO18-29 engaged VP3 using residues from both heavy and light chains, forming an extensive interaction network comprising 17 hydrogen bonds and 5 salt bridges (Table C in S1 Appendix). These interactions involved residues in VP3 B-B knob (OHN-VP3E58), B-C loop (OHN-VP3D69, OHN-VP3S70, and OHN-VP3D71), βC (OHN-VP3Q76), and E-F loop (OHN-VP3E131 and OHN-VP3K134) interacting with residues in the CDRs and FRs of pO18-29, ordinal HCDR3(VHR102, VHR103, VHN105, VHG107, VHY110, VHY108, VHR111, and VHD112), HCDR1(VHY32), LCDR2(VLG51and VLD53), LCDR1(VLY33), L-FR3(VLR55 and VLN54), and L-FR2(Y50) (Fig 5A-D). By contrast, although pO18-29 recognized a largely overlapping surface on the O/18074 capsid, the FMDV-O18–pO18-29 complex exhibited a reconfigured interaction network, characterized by 25 hydrogen bonds and 7 salt bridges at the interface (Table D in S1 Appendix). The residues in VP3 B-B knob (O18-VP3E58), B-C loop (O18-VP3T68, O18-VP3D69, O18-VP3S70, and O18VP3D71), βC (O18-VP3K73 and O18-VP3Q76), C-D loop(O18-VP3H85), E-F loop (O18-VP3E131 and O18-VP3P132), and VP1 C-terminus(O18-VP1A199) participated in contacts with pO18-29, including HCDR3(VHR103, VHN105, VHY110, VHY116, VHG107, and VHY108), HCDR1(VHN31), LCDR2(VLG51and VLD53), L-FR3(VLR55, VLR61 and VLN54), and L-FR2(Y50) (Fig 5E-H). These data reflected a redistribution of intermolecular interactions rather than an expansion of the antigenic footprint.

**Fig 5:**
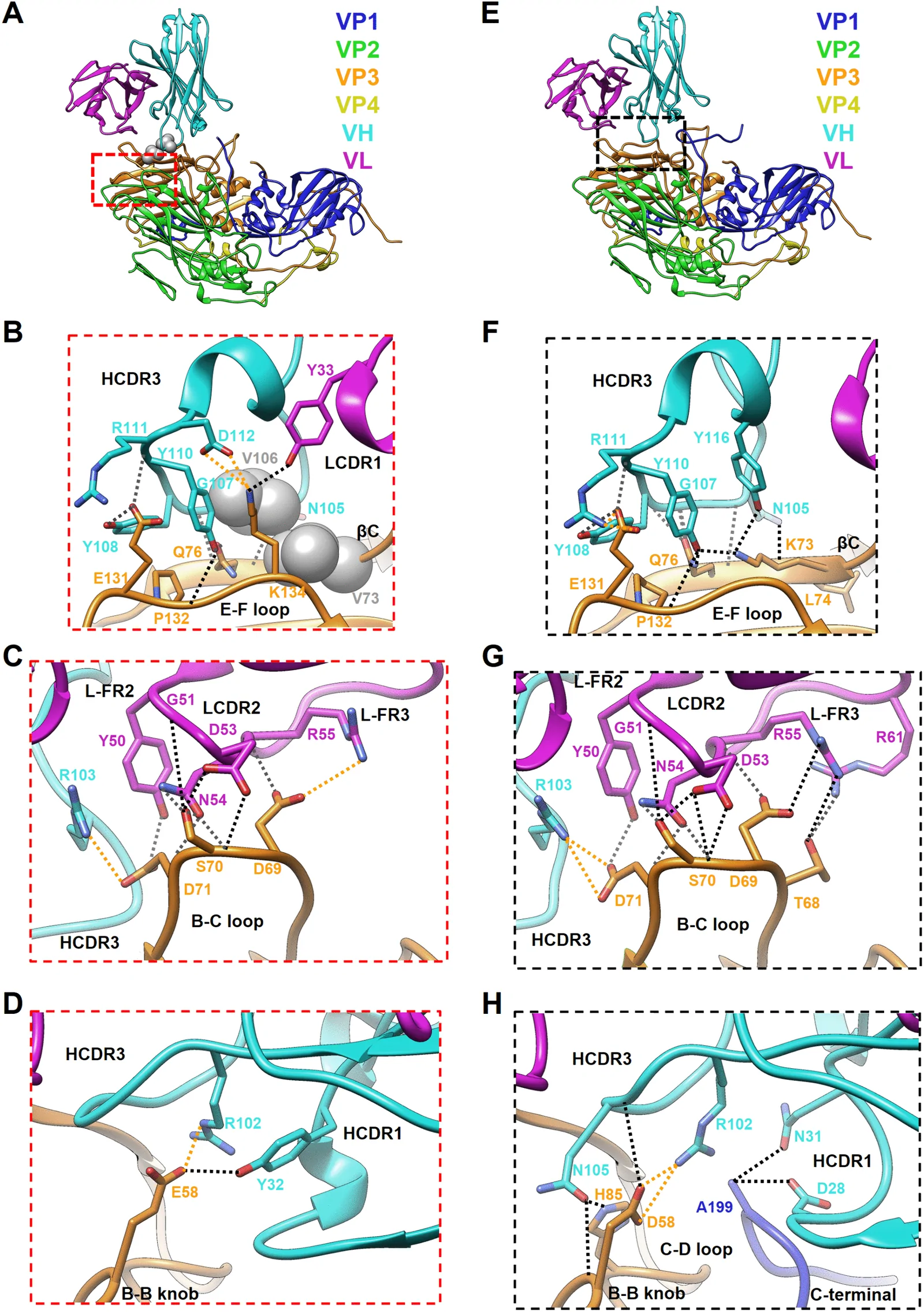
Structural basis of pO18-29 recognition. **(A, E)** Cartoon representations of the interaction interfaces between pO18-29 scFv and the capsids of O/HN/CHA/93 **(A)** and O/18074 **(E)**. The heavy chain and light chain of pO18-29 are colored cyan and magenta, respectively. Capsid proteins VP1 to VP4 are colored blue, green, red and yellow, respectively. **(B-D)** Expanded views of antibody–antigen interface highlight the VP3 E-F loop and βC **(B)**, B-C loop **(C)**, and B-B knob region **(D)** of O/HN/CHA/93. **(F-H)** Expanded views of antibody–antigen interface highlight the VP3 E-F loop and βC **(F)**, B-C loop **(G)**, and B-B knob region **(H)** of O/18074. Putative hydrogen bonds and salt bridges are indicated by black and orange dashed lines, respectively. Hydrophobic interactions are depicted as gray spheres.

Consistent with this interpretation, energetic analysis revealed that, despite similar buried surface areas, the O/HN/CHA/93 complex exhibited higher stabilization and hydrogen-bonding energies than the O/18074 complex (Table E in S1 Appendix). This energetic disparity could be primarily attributed to two local structural differences at the interface (Fig 5). First, VP3 residue K134 in O/HN/CHA/93 formed both hydrogen bonds and salt bridges with pO18-29, whereas the corresponding residue in O/18074 (T134) lacked the positively charged side chain required for salt-bridge formation and instead participated mainly in weaker polar or van der Waals interactions. Second, VP3 residue V73 in O/HN/CHA/93 established a deep hydrophobic interaction with the HCDR3 loop of pO18-29 (Fig 5B), serving as a key anchoring point. This hydrophobic contact was abolished in O/18074 due to the V73K substitution (Fig 5F), resulting in destabilization of the antibody-capsid interface. Collectively, these findings suggest that neutralization escape in the O/18074 variant is driven by subtle remodeling of hydrophobic and electrostatic interactions, leading to reduced interfacial stability without altering the overall antigenic footprint.

### Synergistic mutations at positions 73 (V → K) and 134 (K → T) on VP3 mediate neutralization escape in O/Cathay variants via disruption hydrophobic interaction as well as the hydrogen bonds and salt bridge networks

Cryo-EM analysis revealed that pO18-29 engages a similar antigenic surface on VP3 in both the ancient strain O/HN/CHA/93 and the recent O/Cathay variant O/18074. However, the interaction networks differ. In the O/HN/CHA/93 complex, VP3 residues V73 and K134 formed a dense network of hydrophobic contacts, hydrogen bonds, and salt bridges with the antibody. In contrast, these interactions were substantially weakened in the O/18074 complex due to the V73K and K134T substitutions (Fig 6A), suggesting that residues 73 and 134 may act as key structural determinants of neutralization escape in O/Cathay variants. Consistent with their potential role in neutralization escape, sequence analysis revealed that the V73K substitution became increasingly prevalent among O/Cathay variants after 2015 (Fig B panel B in S1 Appendix), while the K134T substitution showed a marked increase in prevalent strains after 2010 (Fig B panel C in S1 Appendix).

**Fig 6:**
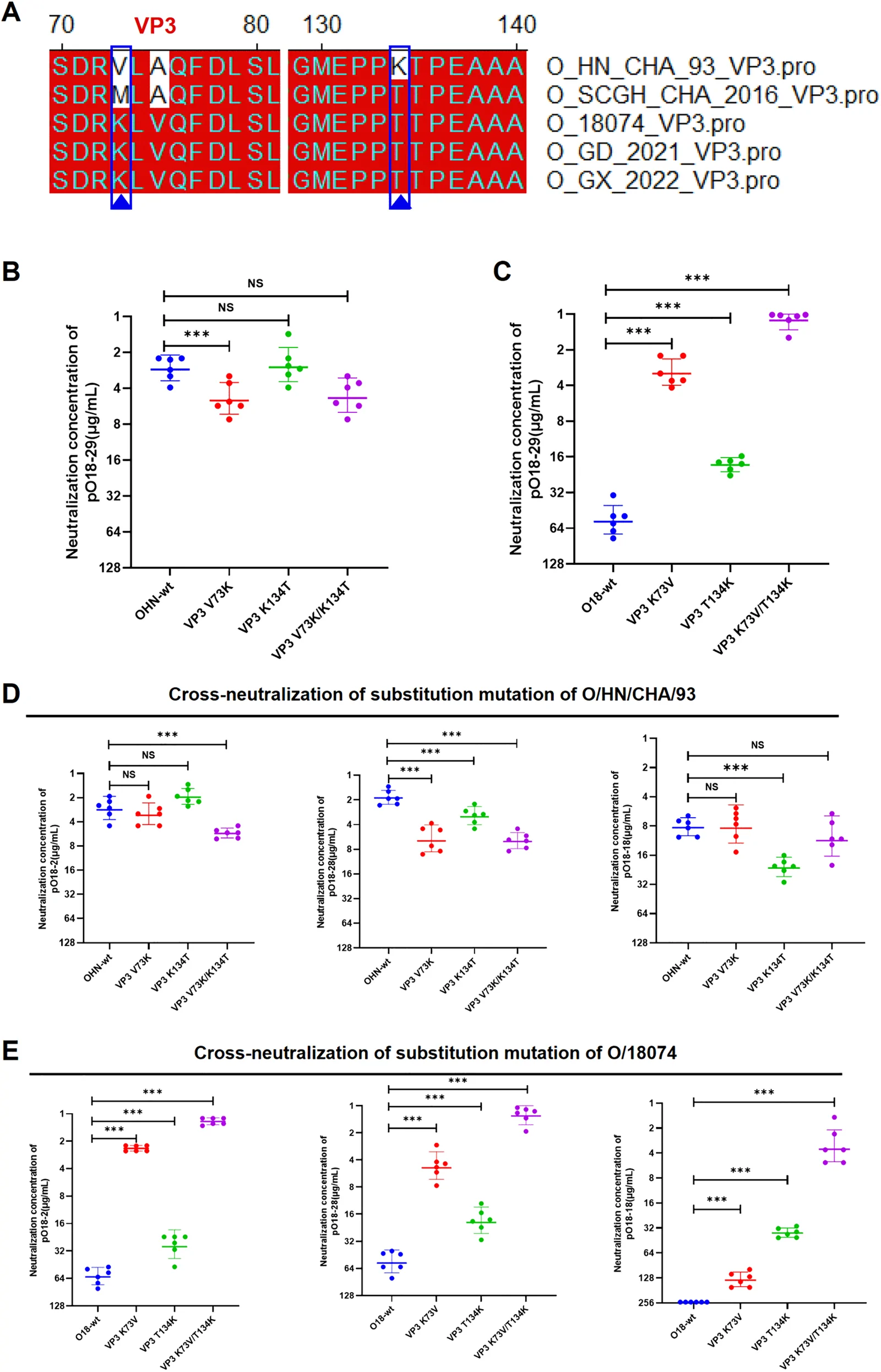
Key VP3 determinants in O/Cathay variants. **(A)** Sequence alignment of VP3 from ancient and variant O/Cathay strains. Residues at positions 73 and 134 are highlighted by blue box. **(B, C)** Neutralizing activity of pO18-29 against wild-type O/HN/CHA/93 (OHN-wt) **(B)** or wild-type O/18074 (O18-wt) **(C)** and the corresponding VP3 mutant viruses, measured using microneutralization assay. The neutralization concentration represented the lowest antibody concentration required to fully prevent CPE. **(D, E)** Neutralizing activity of pnAbs (pO18-2, pO18-28, and pO18-18) against wild-type O/HN/CHA/93 (OHN-wt) **(D)** or wild-type O/18074 (O18-wt) **(E)** and the corresponding VP3 mutant viruses, measured using microneutralization assay. Statistical differences between wild-type and mutant viruses were analyzed using an unpaired *t* test with the Holm–Sidak method (α = 0.05). ***P < 0.001; **P < 0.01; *P < 0.05; NS, not significant. Experiments were performed independently in triplicate.

To functionally validate this hypothesis, we generated recombinant viruses carrying single or double substitutions at VP3 residues 73 and 134 (_OHN-VP3_V73K, _OHN-VP3_K134T, _OHN-VP3_V73K/K134T, _O18-VP3_K73V, _O18-VP3_T134K and _O18-VP3_K73V/T134K) using O/HN/CHA/93 and O/18074 infectious clones. All recombinant viruses were successfully rescued and confirmed by Sanger sequencing and plaque assays (Fig J in S1 Appendix). In the O/HN/CHA/93 background, the V73K substitution markedly reduced neutralization by pO18-29, whereas the K134T substitution alone had minimal effect (Fig 6B). Analysis of additional pnAbs revealed antibody-specific sensitivity patterns. Either the V73K or K134T substitution reduced neutralization by pO18-28, while the combined V73K/K134T mutation impaired neutralization by pO18-2, and the K134T substitution selectively reduced neutralization by pO18-18 (Fig 6D). Notably, in the O/18074 background, the reciprocal K73V or T134K substitutions markedly enhanced neutralization by all four pnAbs, with the combined K73V/T134K substitutions producing the strongest restoration of sensitivity (Fig 6C and 6E). Together, these results identify VP3 residues 73 and 134 as dominant and synergistic determinants of monoclonal antibody-mediated neutralization in O/Cathay strains.

To assess whether these substitutions broadly affected polyclonal sera neutralization, sera from pigs immunized with O/HN/CHA/93 were tested against the recombinant viruses. Neither introduction of the V73K, K134T, or V73K/K134T substitutions in the O/HN/CHA/93 background nor the reciprocal K73V, T134K, or K73V/T134K substitutions in the O/18074 background significantly altered serum neutralization compared with the respective parental viruses (Fig K in S1 Appendix). These findings indicate that VP3 residues 73 and 134 primarily modulate monoclonal antibody recognition and do not substantially affect polyclonal serum neutralization.

In addition to these fixed escape determinants, analysis of neutralization escape mutants selected by pO18-2, pO18-18, pO18-28, and pO18-29 revealed further substitutions clustered in VP3 (Table 1). Escape mutants of pO18-18 predominantly carried double substitutions at residues 71 and 134 (4/8) or 70 and 134 (2/8), with single substitutions at either position being rare (1/8 each). Sanger sequencing of the escape mutants confirmed the presence of these mutations in the viral stocks (Fig L panel A in S1 Appendix). Amplicon sequencing of one representative double mutant further revealed an additional substitution at VP3 residue 70 (Q → R) with a mutation frequency of 0.225 (Fig L panel B in S1 Appendix). Sequence alignment confirmed divergence at VP3 residue 134 between O/HN/CHA/93 and circulating O/Cathay variants, while residue 71 remained highly conserved (Fig M panel A in S1 Appendix). Recombinant viruses carrying single and the combined mutations at positions 71 and 134 were successfully rescued in the O/HN/CHA/93 background (Fig M panels B-C in S1 Appendix). Functional analysis showed that the D71N substitution alone did not affect neutralization by pO18-18, while the K134T substitution markedly reduced neutralization, the combined D71N/K134 mutations completely escape neutralization by pO18-18 (Fig M panel D in S1 Appendix).

Escape mutants selected by pO18‑2 and pO18‑28 exhibited diverse mutation profiles, with substitutions at VP3 residues 70, 76, and 134 appearing most frequently. In contrast, escape mutants selected by pO18-29 consistently carried substitutions at VP3 residues 70 and 76, most frequently in the form of double substitutions (S70P/Q76R, S70L/Q76R, or S70A/Q76R). Sanger sequencing of the escape mutants confirmed the presence of these mutations in the viral stocks (Figs N-P in S1 Appendix). Amplicon sequencing of a pO18‑2 escape mutant carrying Q76R revealed additional mutations at VP3 residue 134 (K → E, 0.333; K → T, 0.414). Representative pO18‑28 and pO18‑29 mutants were also confirmed by amplicon sequencing, with only low‑frequency variants (<1%) detected at other positions (Fig Q in S1 Appendix). Recombinant viruses bearing single or combined mutations at positions 70 and 76 were generated and successfully rescued in the O/HN/CHA/93 background (Fig R panels A-B in S1 Appendix). Single substitutions at residue 70 had no detectable effect on neutralization, while the Q76R substitution conferred partial escape, and the combined S70P/Q76R substitution completely abolished neutralization by pO18-2 and pO18-29(Fig R panel C in S1 Appendix). Despite their functional impact *in vitro*, residues 70 and 76 remained fully conserved among O/HN/CHA/93 and circulating O/Cathay variants (Fig R panel D in S1 Appendix). Together, these data indicate that synergistic mutations facilitate escape from antibody neutralization. The VP3 residues 73 and 134 represent the dominant determinants of antigenic variation in circulating O/Cathay strains, whereas substitutions at residues 70 and 76 contribute to escape only under *in vitro* selective pressure.

## Discussion

In this study, we demonstrated that recent O/Cathay variants exhibit pronounced neutralization escape from multiple serotype O vaccine-induced sera as well as seven host-derived monoclonal neutralizing antibodies. We identified two spatially and mechanistically distinct antigenic regions on the viral capsid that contribute to antigenic variation and neutralization escape of O/Cathay viruses. The crucial antigenic determinant, located at residue 149 (RGD + 2) on the VP1 G-H loop downstream of the conserved RGD receptor-binding motif, modulates neutralization by pnAbs and vaccine-induced sera. In parallel, cryo-EM analysis uncovered a previously uncharacterized antigenic structure mapped to the βC and E-F loops of VP3. The key determinants, residues 73 (V → K) and 134 (K → T), mediate neutralization escape by perturbing hydrophobic contacts and reorganizing hydrogen bond and salt bridge networks. These two residues act synergistically to regulate pnAbs recognition. Comparable immune escape strategies driven by subtle electrostatic and structural remodeling at antibody-binding interfaces have also been reported in other RNA viruses, such as SARS-CoV-2 [24,25] and enteroviruses [26].

FMDV VP1 is a highly variable and immunogenic protein and contains the integrin-binding RGD motif within the G-H loop [27]. As the most conformationally flexible region on the viral surface, the VP1 G-H loop plays a central role in receptor engagement and antigenic variation [28]. Amino acid substitutions within the VP1 G-H loop can markedly alter viral antigenicity and promote escape from neutralizing antibodies [29,30]. Residues flanking the RGD motif are particularly critical for neutralization sensitivity, and coordinated changes at the RGD + 1, RGD + 2 or RGD + 3 positions contribute to fine-tuning the local conformation of the G-H loop [12,31,32]. Such substitutions can modulate antibody recognition without severely compromising viral fitness, underscoring their importance in immune-driven selection [12,33]. Our study identified that the Q149H substitution (RGD + 2) completely abolished neutralization by pnAbs pO18-34, pO18-40, and pO18-43. Reversion of this residue to the ancient amino acid (H149Q) restored sensitivity to serum neutralization. Notably, the Q149H substitution introduces changes in amino acid polarity that are likely to destabilize local loop conformations, thereby altering antigen-antibody interactions. Together, these findings highlight the RGD + 2 position as a critical determinant shaping the antigenic architecture of VP1 and facilitating immune evasion in O/Cathay lineages.

Among the ﬁve known antigen sites of FMDV serotype O, site 4 was deﬁned by mutations at positions 56 and 58 on the VP3 B-B knob by both murine [34,35] and bovine [36] mAbs. Subsequently, a novel antigenic site on VP3, including the βB (T65), B-C loop (T68), E-F loop (E131 and K134), and H-I loop (G196), was identified and shown to be functionally independent of antigenic site 4 by bovine mAbs (C4 and C4-like antibodies) [37]. In our study, we further employed cryo-EM to resolve a previously unrecognized antigenic epitope on VP3, with key residues located at βC (73) and E-F loop (134). Notably, these residues exhibited synergistic effects in mediating neutralization escape of Cathay variants from pnAbs. These changes on VP3 are expected to reduce antibody affinity rather than abolish binding entirely. The cluster’s role as a thermodynamic anchor maximizes the benefit of both van der Waals contacts and the surrounding hydrogen-bond network. In contrast, the mutant interface of O/18074, reliant solely on an increased number of hydrogen bonds without the advantage of a desolvated core, achieves lower affinity due to the energetic penalty associated with hydrogen bond formation in a hydrated environment [38,39].

Viral antigenic evolution or drift is driven by the continual emergence of variants capable of evading the existing immune response [40]. This process reflects a balance between immune selection, structural constraints, and viral fitness [41]. Escape mutations selected in vitro may not fully recapitulate those arising during natural infection; nevertheless, they delineate structurally and antigenically permissible evolutionary trajectories under sustained antibody pressure. Notably, although VP3 residues 70 and 76 are conserved between the ancient O/HN/CHA/93 strain and currently circulating Cathay variants, our mutagenesis data demonstrated that substitutions at these positions can cooperatively mediate escape from antibody neutralization. These findings suggest that this region constitutes a latent antigenic site that is structurally permissive to escape mutations, even though such changes have not yet been fixed in natural populations. Continued immune pressure may therefore unmask the antigenic relevance of this region during future viral evolution.

In summary, this study defines key molecular and structural determinants underlying antigenic variation in the O/Cathay topotype of FMDV. Our findings indicate that substitutions in VP1 and VP3 can reshape dominant neutralizing epitopes and promote immune escape without overt loss of viral viability. These findings provide a mechanistic framework for understanding ongoing antigenic drift in porcinophilic FMDV strains and offer important insights for rational vaccine updating to improve protection against emerging O/Cathay variants in swine populations. Specifically, the identification of VP1 residue 149 (RGD + 2) and VP3 residues 73 and 134 as critical determinants of neutralization escape provides clear targets for monitoring antigenic drift in the field, potentially guiding strain selection for conventional vaccine updates. In addition, the structural characterization of these epitopes may facilitate the design of subunit immunogens that help broaden vaccine‑induced immunity.

## Materials and methods

### Expression and purification of porcine-derived mAbs

The selected paired VH and VL genes were synthesized with codon optimization and respectively cloned into porcine heavy chain (CH-pcDNA3.4) and light chain (Cκ-pcDNA3.4 or Cλ-pcDNA3.4) expression vectors to obtain the antibody-expressing plasmids VH-CH-pcDNA3.4 and VL-Cκ-pcDNA3.4/or VL-Cλ-pcDNA3.4, as described in our previous report [42]. The antibody-expressing plasmids were co-transfected into suspension CHO-S cells (Invitrogen, USA) at a heavy-to-light chain plasmid ratio of 2:3 using the ExpiFectamin CHO Transfection Kit (Invitrogen), according to the manufacturer’s instructions. Enhancer and feed components were added 16 h post-transfection. Antibody-containing supernatants were harvested 8 days after transfection. The supernatants were initially purified through Ni-chelating affinity chromatography. The concentrations of expressed mAbs were determined by measuring absorbance values at 280 nm (A280).

### Indirect immunofluorescence assay

BHK-21 cells in 24-well plates were infected with O/HN/CHA/93 strain of FMDV serotype O at a multiplicity of infection (MOI) of 1.0 for 4h at 37 °C in the presence of 5% CO_2_. Virus-infected cells were then fixed in precooled methanol-acetone solution at a ratio of 1:1 (v/v) for 30 min at 4 °C. After rinsing with PBS, cells were blocked for 1h at 37°C with 1% bovine serum albumin (BSA) in PBS. Then 200 µl per well porcine mAbs at a working concentration of 5µg/mL were added and incubated for 1h at 37°C. After washing with PBS, anti-pig IgG (whole molecule)-FITC antibody (Sigma Aldrich) at a 1:200 dilution was added and incubated in the dark for 1h at 37°C. Plates were washed three times with PBS and observed under the EVOS FL Imaging System (Life Technology, USA).

### Bio-layer interferometry (BLI) assay

Prior to the BLI assay, purified O/HN/CHA/93 and O/18074 viral particles were respectively labeled with EZ-Link Sulfo-NHS-LC-LC-Biotin (Thermo Fisher Scientific, USA) according to the manufacturer’s instructions and then purified using Zeba spin desalting column (Thermo Fisher Scientific) to remove excess non-reacted biotin. To determine the binding affinities of the porcine nAbs to FMDV, BLI measurements were performed in an Octet RED96 System according to manufacturer’s guidelines. Briefly, biotinylated O/HN/CHA/93 or O/18074 virion (10 µg/mL) was respectively immobilized onto streptavidin-coated biosensors (Pall FortéBio) until saturation. The antigen-bound biosensors were then transferred to wells containing serial dilutions of porcine nAbs to monitor the association phase, followed by immersion in dissociation buffer (0.01 M PBS supplemented with 0.1% bovine serum albumin and 0.02% Tween 20) to record dissociation kinetics. The on-rate (ka) and off-rate (kd) were determined by global fitting of the association and dissociation phases of a series of porcine nAbs concentrations. The equilibrium dissociation constant (KD), a measure of affinity, was then calculated as the ratio of kd and ka.

### Virus neutralizing test

The neutralizing activities of porcine monoclonal antibodies (mAbs) and porcine sera were evaluated against seven representative strains of FMDV serotype O, three representative strains of serotype A, and the rescued virus using a micro-neutralization assay as previously described [43]. The porcine sera were obtained from four immunization groups vaccinated with inactivated vaccines containing 146S antigens derived from O/HN/CHA/93 (Cathay), O/XJ/CHA/2017 (O/Ind/2001 lineage), O/Tibet/99 (O/PanAsia lineage), or O/GSLX/2010 (O/Mya98 lineage). The FMDV serotype O panel included O/Tibet/99 and O/GZ1/2025 (ME-SA topotype); O/GSLX/2010 and O/HNNY/2022 (SEA topotype), O/HN/CHA/93 (Cathay ancient strain); and O/SCGH/CHA/2016, O/18074, O/GD/2021, O/GX/2022 (Cathay variants). The serotype A panel included A/AF72 strain (A22 lineage), A/WH/CHA/09 and A/GDMM/2013 strains (Sea97 lineage). All virus strains used in this study are listed in Table F in S1 Appendix. Briefly, antibody samples were 2-fold serially diluted in 96-well cell culture plates in a total volume of 50 µl, and then 100 TCID_50_ of FMDV in 50 µl of culture media was added to each well. After incubation for 1h at 37 °C, approximately 5 × 10^4^ BHK-21 cells in 100 µl culture media were added to each well as indicators of residual infectivity. Normal cell wells, and virus control wells (0.1, 1, 10 and 100TCID_50_) in duplicate were used in each plate for comparison purpose. The plates were incubated at 37 °C under 5% CO_2_ conditions for 72h before observing cytopathic effect (CPE). The experimental results were acceptable when complete CPE and no CPE appeared separately in 100TCID_50_ and 0.1TCID_50_ virus control wells. The endpoint titers were calculated as the reciprocal of the last antibody dilution to neutralize 100TCID_50_ FMDV in 50% of the wells. Neutralizing activity was expressed as the virus neutralization (VN) titer, calculated as the initial antibody concentration divided by the endpoint dilution.

### Selection of neutralization-escape mutants using porcine neutralizing antibodies

Neutralization escape mutants were generated by serial passaging of FMDV in BHK-21 cells under the selective pressure of pnAbs, following a previously described method with minor modifications [44]. The representative FMDV strain O/HN/CHA/93 was used for mutant selection. Briefly, 10-fold serial dilutions of FMDV in 50 μl were incubated with 50 μl of various concentrations of mAbs (10 μg/mL to 20 μg/mL) in 96-well cell culture plates. The mixtures were used to infect BHK-21 cells (10^6^ cells/mL) in a volume of 100 μl and incubated at 37˚C for 48 h to allow virus propagation. The first-passage viruses were harvested from the wells seeded with the highest dilution of virus that produced an approximately 80% to 100% cytopathic effect (CPE). Subsequent rounds of selection were performed in 24-well plates. In each round, the passaged virus (200 μl) was incubated with an equal volume of 2-fold concentration of antibodies in each well containing BHK-21 cells (400 μl). Process was repeated for multiple passages until viral replication was observed in the presence of mAbs at concentrations exceeding 400 μg/mL, indicating complete escape from antibody neutralization. For each antibody, four escape mutants (A-D) were selected using an initial mAb concentration of 10 μg/ml, and four additional mutants (E-H) were selected using 20 μg/ml. The A–D mutants were serially passaged for seven rounds up to 640 μg/ml, whereas the E–H mutants were passaged for six rounds to reach the same final concentration. Viral RNA was then extracted, and the P1 region of the escape mutants was amplified by one-step RT-PCR as previously described [45], employing the primer pair Pan204+(ACCTCCAACGGGTGGTACGC) /NK61 (GACATGTCCTCTTGCATCTG). A portion of amplified products were verified by Sanger sequencing. Mutated amino acids were determined by aligning the entire mutant P1 region with its parent virus sequence.

### Amplicon sequencing and data analysis

To quantify the mutation frequencies in the viral stocks, the remaining PCR products from parent virus and seven representative escape mutants (one per pnAb) were subjected to agarose gel electrophoresis, and the target bands were excised and purified using a gel extraction kit. The purified amplicons were then sent to GENEWIZ (Azenta Life Sciences, Suzhou, China) for amplicon sequencing on an Illumina platform. The raw sequencing data were generated as FASTQ files (PF data or Raw data). Quality control and data analysis were performed to identify mutations relative to the parental O/HN/CHA/93 sequence and to calculate the allele frequency of each variant. Reads were quality-filtered and aligned to the parental reference sequence, and variants with a frequency above the detection threshold were reported.

### Rescue and titration of site-directed FMDV mutants by reverse genetics

Full-length cDNAs were generated using an established pOFS plasmid containing the complete P1 gene of FMDV strain O/HN/CHA/93. Site-directed mutagenesis was applied to introduce nucleic acid mutations to produce full-length cDNAs with single amino acid substitutions [46]. All mutant constructs were confirmed by Sanger sequencing. Recombinant FMDV mutants were rescued using a reverse genetics system as previously reported [4]. Briefly, mutant plasmids were linearized with *NotI* and transfected into BSR/T7 cells using Lipofectamine 2000 (Invitrogen) according to the manufacturer’s instructions. The transfected cells were monitored daily for CPE appearance. At 72 h post-transfection, the culture supernatants and cells were harvested and passaged up to the fifth passage on BHK-21 cells. Viral RNA was then extracted, and the P1 region of the mutants was amplified by one-step RT-PCR, employing the primer pair Pan204+(ACCTCCAACGGGTGGTACGC) /NK61 (GACATGTCCTCTTGCATCTG). The amplified products were confirmed by Sanger sequencing. Mutated amino acids were identified by aligning the entire mutant P1 region with its parent virus sequence. The fifth-passage rescued mutant viruses were measured using the plaque forming unit (PFU) assay. Confluent monolayers of BHK-21 cells were infected with 10-fold serially diluted FMDV samples in 6-well plates. Tragacanth gum (0.6%) was added after 1 h of incubation. Plaques were visualized at 48 h postinfection by fixing with acetone-methanol and staining with crystal violet. The mutant virus titers were further quantified in BHK-21 cells by calculating the 50% tissue culture infectious dose (TCID_50_), subsequently used to perform micro-neutralization assay as described above.

### Cryo-EM sample preparation, data collection and three-dimensional reconstruction

Purified FMDV O/HN/CHA/93 146S and O/18074 146S particles were individually incubated with scFv at a molar ratio of 1:240 in a volume of 10 μl for 10–60 mins at 4˚C. A 4-μl aliquot of the mixture was applied to a glow-discharged Quantifoil grid (Au 1.2/1.3, 200 mesh). Grids were blotted for 3 s at 100% relative humidity and plunge-frozen in liquid ethane using a Vitrobot Mark IV (Thermo Fisher, USA). Cryo-EM data were collected on a Titan Krios G3i transmission electron microscope (Thermo Fisher Scientific) operated at 300 kV and equipped with a K3 BioQuantum direct electron detector (Gatan) at Lanzhou University. Micrograph images were collected as movies (15 frames, 1.5 s) and recorded at −2.0 to −0.8 μm under focus at a calibrated magnification of ×105 kX, resulting in a pixel size of 0.83 Å.

The raw movie stacks were imported into cryoSPARC [47] for image processing and three-dimensional (3D) reconstruction. Individual frames from each micrograph movie were aligned and averaged to produce drift-corrected images. Particles were automatically picked and selected, and contrast transfer function (CTF) parameters were estimated using CTFFIND4 [48]. Subsequent 3D reconstruction steps, including ab initio model generation, heterogeneous refinement, and homogeneous refinement, were performed in cryoSPARC. Final map resolutions were determined using the gold-standard Fourier shell correlation (FSC) criterion at 0.143. The data collection and refinement statistics are summarized in Table G in S1 Appendix.

### Model building and data analysis

To construct initial atomic models of FMDV-OHN-pO18-29 and FMDV-O18-pO18-29 complexes, the cryo-EM structure of native FMDV-OTi (PDB:7D3K) [15] and the AlphaFold2-predicted structure of pO18-29 [49] were manually placed into the corresponding cryo-EM density maps and rigid-body fitted with UCSF Chimera [50]. The resulting complex models were subsequently refined by real-space refinement in Phenix [51]. Manual model building was performed using Coot [52], followed by additional rounds of real-space refinement in Phenix to improve the agreement between the models and the density maps. The final models were validated using MolProbity [53].

## Supporting information

S1 Appendix Suppementary figures and tables. (DOCX)

## References

[1] Knight-Jones TJD, Rushton J. The economic impacts of foot and mouth disease - what are they, how big are they and where do they occur? Prev Vet Med. 2013;112(3–4):161–73. doi: 10.1016/j.prevetmed.2013.07.013 23958457 PMC3989032

[2] Knowles NJ, Samuel AR. Molecular epidemiology of foot-and-mouth disease virus. Virus Res. 2003;91(1):65–80. doi: 10.1016/s0168-1702(02)00260-5 12527438

[3] Mahapatra M, Parida S. Foot and mouth disease vaccine strain selection: current approaches and future perspectives. Expert Rev Vaccines. 2018;17(7):577–91. doi: 10.1080/14760584.2018.1492378 29950121

[4] Li P, Bai X, Sun P, Li D, Lu Z, Cao Y, et al. Evaluation of a genetically modified foot-and-mouth disease virus vaccine candidate generated by reverse genetics. BMC Vet Res. 2012;8:57. doi: 10.1186/1746-6148-8-57 22591597 PMC3488552

[5] Upadhyaya S, Mahapatra M, Mioulet V, Parida S. Molecular Basis of Antigenic Drift in Serotype O Foot-and-Mouth Disease Viruses (2013-2018) from Southeast Asia. Viruses. 2021;13(9):1886. doi: 10.3390/v13091886 34578467 PMC8473337

[6] Li F, Li Y, Ma J, Wu R, Zou X, Liu Y, et al. Molecular evolution, diversity, and adaptation of foot-and-mouth disease virus serotype O in Asia. Front Microbiol. 2023;14:1147652. doi: 10.3389/fmicb.2023.1147652 36970668 PMC10034406

[7] Zhu Z, Yang F, Cao W, Liu H, Zhang K, Tian H, et al. The Pseudoknot Region of the 5’ Untranslated Region Is a Determinant of Viral Tropism and Virulence of Foot-and-Mouth Disease Virus. J Virol. 2019;93(8):e02039-18. doi: 10.1128/JVI.02039-18 30728251 PMC6450104

[8] Knowles NJ, Davies PR, Henry T, O’Donnell V, Pacheco JM, Mason PW. Emergence in Asia of foot-and-mouth disease viruses with altered host range: characterization of alterations in the 3A protein. J Virol. 2001;75(3):1551–6. doi: 10.1128/JVI.75.3.1551-1556.2001 11152528 PMC114061

[9] Hui RK, Leung FC. Evolutionary trend of foot-and-mouth disease virus in Hong Kong. Vet Microbiol. 2012;159(1–2):221–9. doi: 10.1016/j.vetmic.2012.03.016 22472703

[10] Dunn CS, Donaldson AI. Natural adaption to pigs of a Taiwanese isolate of foot-and-mouth disease virus. Vet Rec. 1997;141(7):174–5. doi: 10.1136/vr.141.7.174 9290197

[11] Beard CW, Mason PW. Genetic determinants of altered virulence of Taiwanese foot-and-mouth disease virus. J Virol. 2000;74(2):987–91. doi: 10.1128/jvi.74.2.987-991.2000 10623761 PMC111619

[12] Li F, Wu S, Lv L, Huang S, Zhang Z, Zerang Z, et al. Discovery, recognized antigenic structures, and evolution of cross-serotype broadly neutralizing antibodies from porcine B-cell repertoires against foot-and-mouth disease virus. PLoS Pathog. 2024;20(10):e1012623. doi: 10.1371/journal.ppat.1012623 39405339 PMC11508087

[13] Li P, et al. Evaluation of immunogenicity and cross-reactive responses of vaccines prepared from two chimeric serotype O foot-and-mouth disease viruses in pigs and cattle. Vet Res. 2022;53(1):56. 10.1186/s13567-022-01072-7 PMC9270804 35804412

[14] Di Nardo A, Knowles NJ, Wadsworth J, Haydon DT, King DP. Phylodynamic reconstruction of O CATHAY topotype foot-and-mouth disease virus epidemics in the Philippines. Vet Res. 2014;45(1):90. doi: 10.1186/s13567-014-0090-y 25209700 PMC4177241

[15] Mattion N, Goris N, Willems T, Robiolo B, Maradei E, Beascoechea CP, et al. Some guidelines for determining foot-and-mouth disease vaccine strain matching by serology. Vaccine. 2009;27(5):741–7. doi: 10.1016/j.vaccine.2008.11.026 19041355

[16] Nishi T, Morioka K, Kawaguchi R, Yamada M, Ikezawa M, Fukai K. Quantitative analysis of infection dynamics of foot-and-mouth disease virus strain O/CATHAY in pigs and cattle. PLoS One. 2021;16(1):e0245781. doi: 10.1371/journal.pone.0245781 33481934 PMC7822254

[17] Lea S, Abu-Ghazaleh R, Blakemore W, Curry S, Fry E, Jackson T, et al. Structural comparison of two strains of foot-and-mouth disease virus subtype O1 and a laboratory antigenic variant, G67. Structure. 1995;3(6):571–80. doi: 10.1016/s0969-2126(01)00191-5 8590018

[18] Jinding C, Mingqiu Z, Hui KH, Leung FC. Molecular characterization of foot-and-mouth disease virus in Hong Kong during 2001-2002. Virus Genes. 2006;32(2):139–43. doi: 10.1007/s11262-005-6869-1 16604445

[19] Gleeson LJ. A review of the status of foot and mouth disease in South-East Asia and approaches to control and eradication. Rev Sci Tech. 2002;21(3):465–75. doi: 10.20506/rst.21.3.1346 12530354

[20] Upadhyaya S, Mahapatra M, Mioulet V, Parida S. Molecular Basis of Antigenic Drift in Serotype O Foot-and-Mouth Disease Viruses (2013-2018) from Southeast Asia. Viruses. 2021;13(9):1886. doi: 10.3390/v13091886 34578467 PMC8473337

[21] Lee H-W, Deng M-C, Pan C-H, Chang H-W, Cheng I-C. Neutralizing monoclonal antibodies against porcinophilic foot-and-mouth disease virus mapped to antigenic site 2 by utilizing novel mutagenic virus-like particles to detect the antigenic change. Vet Microbiol. 2018;222:124–31. doi: 10.1016/j.vetmic.2018.06.002 30080667

[22] von Buch KG, Engelking R, Heberer G. Aortic coarctation (aortic isthmus stenosis) in infancy and childhood. Dtsch Med Wochenschr. 1966;91(41):1827–32. 5928173

[23] Monto AS, et al. The Doctrine of Original Antigenic Sin: Separating Good From Evil. J Infect Dis. 2017;215(12):1782–8. 10.1093/infdis/jix173 PMC5853211 28398521

[24] Harvey WT, Carabelli AM, Jackson B, Gupta RK, Thomson EC, Harrison EM, et al. SARS-CoV-2 variants, spike mutations and immune escape. Nat Rev Microbiol. 2021;19(7):409–24. doi: 10.1038/s41579-021-00573-0 34075212 PMC8167834

[25] Zhang Z, Zhang J, Wang J. Surface charge changes in spike RBD mutations of SARS-CoV-2 and its variant strains alter the virus evasiveness via HSPGs: A review and mechanistic hypothesis. Front Public Health. 2022;10:952916. doi: 10.3389/fpubh.2022.952916 36091499 PMC9449321

[26] Shakeel S, Westerhuis BM, Domanska A, Koning RI, Matadeen R, Koster AJ, et al. Multiple capsid-stabilizing interactions revealed in a high-resolution structure of an emerging picornavirus causing neonatal sepsis. Nat Commun. 2016;7:11387. doi: 10.1038/ncomms11387 27435188 PMC4961769

[27] Han S-C, Guo H-C, Sun S-Q. Three-dimensional structure of foot-and-mouth disease virus and its biological functions. Arch Virol. 2015;160(1):1–16. doi: 10.1007/s00705-014-2278-x 25377637

[28] Zhang Z, Waters R, Li Y. Pathogenesis of non-epithelial foot-and-mouth disease in neonatal animals. Vet Microbiol. 2021;254:108961. doi: 10.1016/j.vetmic.2020.108961 33545638

[29] Wang H, Xue M, Yang D, Zhou G, Wu D, Yu L. Insertion of type O-conserved neutralizing epitope into the foot-and-mouth disease virus type Asia1 VP1 G-H loop: effect on viral replication and neutralization phenotype. J Gen Virol. 2012;93(Pt 7):1442–8. doi: 10.1099/vir.0.040253-0 22513388

[30] Fernandez-Sainz I, Gavitt TD, Koster M, Ramirez-Medina E, Rodriguez YY, Wu P, et al. The VP1 G-H loop hypervariable epitope contributes to protective immunity against Foot and Mouth Disease Virus in swine. Vaccine. 2019;37(26):3435–42. doi: 10.1016/j.vaccine.2019.05.019 31085001

[31] Li K, He Y, Wang L, Li P, Bao H, Huang S, et al. Conserved antigen structures and antibody-driven variations on foot-and-mouth disease virus serotype A revealed by bovine neutralizing monoclonal antibodies. PLoS Pathog. 2023;19(11):e1011811. doi: 10.1371/journal.ppat.1011811 37983290 PMC10695380

[32] Mahapatra M, Seki C, Upadhyaya S, Barnett PV, La Torre J, Paton DJ. Characterisation and epitope mapping of neutralising monoclonal antibodies to A24 Cruzeiro strain of FMDV. Vet Microbiol. 2011;149(1–2):242–7. doi: 10.1016/j.vetmic.2010.11.003 21144677

[33] Lawrence P, Pacheco JM, Uddowla S, Hollister J, Kotecha A, Fry E, et al. Foot-and-mouth disease virus (FMDV) with a stable FLAG epitope in the VP1 G-H loop as a new tool for studying FMDV pathogenesis. Virology. 2013;436(1):150–61. doi: 10.1016/j.virol.2012.11.001 23228860

[34] Crowther JR, Farias S, Carpenter WC, Samuel AR. Identification of a fifth neutralizable site on type O foot-and-mouth disease virus following characterization of single and quintuple monoclonal antibody escape mutants. J Gen Virol. 1993;74 (Pt 8):1547–53. doi: 10.1099/0022-1317-74-8-1547 8393912

[35] Kitson JD, McCahon D, Belsham GJ. Sequence analysis of monoclonal antibody resistant mutants of type O foot and mouth disease virus: evidence for the involvement of the three surface exposed capsid proteins in four antigenic sites. Virology. 1990;179(1):26–34. doi: 10.1016/0042-6822(90)90269-w 1699353

[36] Barnett PV, Samuel AR, Pullen L, Ansell D, Butcher RN, Parkhouse RM. Monoclonal antibodies, against O1 serotype foot-and-mouth disease virus, from a natural bovine host, recognize similar antigenic features to those defined by the mouse. J Gen Virol. 1998;79 (Pt 7):1687–97. doi: 10.1099/0022-1317-79-7-1687 9680132

[37] Li K, He Y, Wang L, Li P, Wang S, Sun P, et al. Two Cross-Protective Antigen Sites on Foot-and-Mouth Disease Virus Serotype O Structurally Revealed by Broadly Neutralizing Antibodies from Cattle. J Virol. 2021;95(21):e0088121. doi: 10.1128/JVI.00881-21 34406868 PMC8513477

[38] Kathuria SV, Chan YH, Nobrega RP, Özen A, Matthews CR. Clusters of isoleucine, leucine, and valine side chains define cores of stability in high-energy states of globular proteins: Sequence determinants of structure and stability. Protein Sci. 2016;25(3):662–75. doi: 10.1002/pro.2860 26660714 PMC4815418

[39] Basak S, Nobrega RP, Tavella D, Deveau LM, Koga N, Tatsumi-Koga R, et al. Networks of electrostatic and hydrophobic interactions modulate the complex folding free energy surface of a designed βα protein. Proc Natl Acad Sci U S A. 2019;116(14):6806–11. doi: 10.1073/pnas.1818744116 30877249 PMC6452746

[40] Nowak MA. Variability of HIV infections. J Theor Biol. 1992;155(1):1–20. doi: 10.1016/s0022-5193(05)80545-4 1619947

[41] Di Nardo A, Ferretti L, Wadsworth J, Mioulet V, Gelman B, Karniely S, et al. Evolutionary and Ecological Drivers Shape the Emergence and Extinction of Foot-and-Mouth Disease Virus Lineages. Mol Biol Evol. 2021;38(10):4346–61. doi: 10.1093/molbev/msab172 34115138 PMC8476141

[42] Li K, Zhu G, Zhou S, Sun P, Wang H, Bao H, et al. Isolation and characterization of porcine monoclonal antibodies revealed two distinct serotype-independent epitopes on VP2 of foot-and-mouth disease virus. J Gen Virol. 2021;102(7):10.1099/jgv.0.001608. doi: 10.1099/jgv.0.001608 34280085

[43] Li K, Wang S, Cao Y, Bao H, Li P, Sun P, et al. Development of Foot-and-Mouth Disease Virus-Neutralizing Monoclonal Antibodies Derived From Plasmablasts of Infected Cattle and Their Germline Gene Usage. Front Immunol. 2019;10:2870. doi: 10.3389/fimmu.2019.02870 31867017 PMC6908506

[44] Xie QC, McCahon D, Crowther JR, Belsham GJ, McCullough KC. Neutralization of foot-and-mouth disease virus can be mediated through any of at least three separate antigenic sites. J Gen Virol. 1987;68(Pt 6):1637–47. doi: 10.1099/0022-1317-68-6-1637 2438378

[45] Bai X, Bao H, Li P, Sun P, Kuang W, Cao Y, et al. Genetic characterization of the cell-adapted PanAsia strain of foot-and-mouth disease virus O/Fujian/CHA/5/99 isolated from swine. Virol J. 2010;7:208. doi: 10.1186/1743-422X-7-208 20807416 PMC2939563

[46] Bai XW, et al. Engineering Responses to Amino Acid Substitutions in the VP0- and VP3-Coding Regions of PanAsia-1 Strains of Foot-and-Mouth Disease Virus Serotype O. J Virol. 2019;93(7). 10.1128/JVI.02278-18 PMC6430551 30700601

[47] Punjani A, Rubinstein JL, Fleet DJ, Brubaker MA. cryoSPARC: algorithms for rapid unsupervised cryo-EM structure determination. Nat Methods. 2017;14(3):290–6. doi: 10.1038/nmeth.4169 28165473

[48] Mindell JA, Grigorieff N. Accurate determination of local defocus and specimen tilt in electron microscopy. J Struct Biol. 2003;142(3):334–47. doi: 10.1016/s1047-8477(03)00069-8 12781660

[49] Jumper J, Evans R, Pritzel A, Green T, Figurnov M, Ronneberger O, et al. Highly accurate protein structure prediction with AlphaFold. Nature. 2021;596(7873):583–9. doi: 10.1038/s41586-021-03819-2 34265844 PMC8371605

[50] Pettersen EF, Goddard TD, Huang CC, Couch GS, Greenblatt DM, Meng EC, et al. UCSF Chimera--a visualization system for exploratory research and analysis. J Comput Chem. 2004;25(13):1605–12. doi: 10.1002/jcc.20084 15264254

[51] Liebschner D, Afonine PV, Baker ML, Bunkóczi G, Chen VB, Croll TI, et al. Macromolecular structure determination using X-rays, neutrons and electrons: recent developments in Phenix. Acta Crystallogr D Struct Biol. 2019;75(Pt 10):861–77. doi: 10.1107/S2059798319011471 31588918 PMC6778852

[52] Emsley P, Lohkamp B, Scott WG, Cowtan K. Features and development of Coot. Acta Crystallogr D Biol Crystallogr. 2010;66(Pt 4):486–501. doi: 10.1107/S0907444910007493 20383002 PMC2852313

[53] Williams CJ, Headd JJ, Moriarty NW, Prisant MG, Videau LL, Deis LN, et al. MolProbity: More and better reference data for improved all-atom structure validation. Protein Sci. 2018;27(1):293–315. doi: 10.1002/pro.3330 29067766 PMC5734394

[54] Xiao C, Rossmann MG. Interpretation of electron density with stereographic roadmap projections. J Struct Biol. 2007;158(2):182–7. doi: 10.1016/j.jsb.2006.10.013 17116403 PMC1978246

