# Supplementary material for: Structural basis of antigenic drift in FMDV O/Cathay topotype variants revealed by porcine-derived single B-cell neutralizing antibodies
Source: PLoS Pathog. 2026 Oct 15;22(10):e1014650. doi: 10.1371/journal.ppat.1014650 (PMC13671523; doi:10.1371/journal.ppat.1014650)
Supplement: Supplementary file 1 [file ppat.1014650.s001.docx]

## **Supporting Information for**

## **Structural basis of antigenic drift on FMDV O/Cathay topotype variants revealed by porcine-derived single B-cell neutralizing antibodies**

## **Author**

Fengjuan Li^1,3#^, Shanquan Wu^2#^, Shulun Huang^1,3#^, Lv Lv^1,3^, Yimei Cao^1,3^, Huifang Bao^1,3^, Pu Sun^1,3^, Xingwen Bai^1,3^, Jiaxin Yang^1,3^, Jingjing Zha^1,3^, Qiongqiong Zhao^1^, Hehe Zhang^1,3^, Dong Li^1,3^, Jijun He^1,3^, Zaixin Liu^1,3^, Pinghua Li^1,3*^, Zengjun Lu^1,3*^, Dongsheng Lei^2,1,4*^, Kun Li^1,3*^

^1^ State Key Laboratory of Animal Disease Control and Prevention, College of Veterinary Medicine, Lanzhou University, Lanzhou Veterinary Research Institute, Chinese Academy of Agricultural Sciences, Lanzhou 730000, China.

^2^ Key Laboratory of Magnetism and Magnetic Functional Materials, School of Physical Science and Technology, Electron Microscopy Centre of Lanzhou University, Lanzhou University, Lanzhou 730000, China.

^3^ Gansu Province Research Center for Basic Disciplines of Pathogen Biology, Lanzhou 730046, China.

^4^ Jiangsu Key Laboratory of Zoonosis, Yangzhou University, Yangzhou 225009, China

^#^These authors contributed equally to this work.

*Correspondence should be sent to: Pinghua Li ([lipinghua@caas.cn](mailto:lipinghua@caas.cn)), Zengjun Lu ([luzengjun@caas.cn](mailto:luzengjun@caas.cn)), Dongsheng Lei (leids@lzu.edu.cn) and Kun Li ([likun02@caas.cn](mailto:likun02@caas.cn)).

**Figures A to R**

**Fig. A. Neutralization of ME-SA (O/GZ1/2025) and SEA (O/HNNY/2022) strains by porcine sera. (A-D)** Neutralizing titers against O/HN/CHA/93, O/GZ1/2025, and O/HNNY/2022 were determined using sera from pigs immunized with inactivated 146S antigens derived from O/HN/CHA/93 (Cathay) **(A)**, O/Tibet/99 (O/PanAsia lineage) **(B)**, O/XJ/CHA/2017 (O/Ind/2001 lineage) **(C)**, or O/GSLX/2010 (O/Mya98 lineage) **(D)**. Vaccine strains and sample sizes (n) are indicated in the panel titles. Symbols indicate fold changes in geometric mean titers relative to the homologous O/HN/CHA/93 strain. Statistical differences were analyzed by one‑way ANOVA followed by Dunnett's multiple‑comparison test. ***P < 0.001; **P < 0.01; *P < 0.05. Experiments were performed in triplicate.

**Fig. B. Phylogenetic analysis of the P1 region of Cathay, ME-SA, and SEA strains. (A)** Phylogenetic tree based on the P1 nucleotide sequences. **(B-C)** Temporal distribution of VP3 residues 73 **(B)** or 134 **(C)** among the strains included in the phylogenetic analysis, shown as percentage frequency.

**Fig. C. Reactivity of porcine mAbs against to O/18074 evaluated by indirect immunofluorescence assay (IFA).** BHK-21 cells infected with the O/18074 strain were incubated with porcine mAbs (5 μg/mL), followed by detection with FITC-conjugated rabbit anti-pig IgG. Images were acquired using a fluorescence imaging system. Experiments were performed independently in triplicate.

**Fig. D. Sanger sequencing chromatograms of parent virus and escape mutants selected by pO18-34.** The P1 region of the parental virus and escape mutants was amplified and sequenced. Only the segment containing the mutated site(s) is shown. Red boxes indicate the positions of the identified substitutions.

**Fig. E. Sanger sequencing chromatograms of parent virus and escape mutants selected by pO18-40.** The P1 region of the parental virus and escape mutants was amplified and sequenced. Only the segment containing the mutated site(s) is shown. Red boxes indicate the positions of the identified substitutions.

**Fig. F. Sanger sequencing chromatograms of parent virus and escape mutants selected by pO18-43.** The P1 region of the parental virus and escape mutants was amplified and sequenced. Only the segment containing the mutated site(s) is shown. Red boxes indicate the positions of the identified substitutions.

**Fig. G.** **Amplicon sequencing analysis of escape mutants selected by pO18-34, pO18-40, and pO18-43.** For each mutant, the figure lists the mutation position (nucleotide and corresponding amino acid), parental reference, mutant sequence, read depth, and allele frequency. The mutated nucleotide is highlighted in red.

**Fig. H. Sanger sequencing chromatograms and plaque phenotypes of site-directed rescued viruses. (A-B)** Sanger sequencing chromatograms of wild-type and VP1-mutant viruses (OHN-wt vs OHN-VP1 Q149H; O18-wt vs O18-VP1 H149Q). Red box indicates the identified substitution. **(C)** Plaque phenotypes of the site-directed rescued viruses.

**Fig. I. Cryo-EM analysis of FMDV-OHN-pO18-29 complex and FMDV-O18-pO18-29 complex. (A, B)** Fourier shell correlation (FSC) curves of the FMDV-OHN-pO18-29 complex **(A)** and the FMDV-O18-pO18-29 complex **(B)**.

**Fig. J. Sanger sequencing chromatograms and plaque phenotypes of site-directed rescued viruses. (A-B)** Sanger sequencing chromatograms of wild-type and VP3-mutant viruses (OHN-wt vs OHN-VP3 V73K, K134T, and V73K/K134T; O18-wt vs O18-VP3 K73V, T134K, and K73V/T134K). Red boxes indicate substitutions. **(C-D)** Plaque phenotypes of the site-directed rescued viruses.

**Fig. K. Neutralization of VP3 mutant viruses by porcine sera** **from O/HN/CHA/93-immunized pigs. (A, B)**Serum neutralization titers of OHN-wt (O/HN/CHA/93) **(A)** or O18-wt (O/18074) **(B)** and their VP3 mutants measured in porcine sera vaccinated with O/HN/CHA/93 antigen. The vaccine strain and sample size (n) are indicated in the panel. Statistical differences were analyzed by one‑way ANOVA followed by Dunnett's multiple‑comparison test. NS, not significant. Experiments were performed independently in triplicate.

**Fig. L.** **Amplicon sequencing analysis of escape mutants selected by pO18-18. (A)** Sanger sequencing chromatograms of parent virus and escape mutants selected by pO18-18. The P1 region of the parental virus and escape mutants was amplified and sequenced. Only the segment containing the mutated site(s) is shown. Red boxes indicate the positions of the identified substitutions. **(B)** Amplicon sequencing analysis of escape mutants selected by pO18-18. For each mutant, the figure lists the mutation position (nucleotide and corresponding amino acid), parental reference, mutant sequence, read depth, and allele frequency. The mutated nucleotide is highlighted in red.

**Fig. M. Identification of key variant residues in O/Cathay strains recognized by porcine mAb pO18-18. (A)** Sequence alignment of VP3 from ancient and variant O/Cathay strains. Residues at positions 71 and 134 in VP3 identified for pO18-18 recognition are highlighted in blue boxes. **(B)** Sanger sequencing chromatograms of rescued VP3 mutant viruses derived from wild-type O/HN/CHA/93, including VP3 D71N, VP3 K134T, and the double mutant VP3 D71N/K134T. **(C)** Plaque phenotypes of rescued VP3 mutant viruses described in **(B)**. **(D)** Neutralizing activity of pO18-18 against wild-type O/HN/CHA/93 and the corresponding VP3 mutant viruses determined by microneutralization assay. The neutralization diffe rences between wild-type and its mutants were determined using an unpaired t test with Holm-Sidak method (α = 0.05) in GraphPad Prism 7.2. ***P < 0.001; **P < 0.01; *P < 0.05; NS, not significant. Experiments were performed independently in triplicate.

**Fig. N. Sanger sequencing chromatograms of parent virus and escape mutants selected by pO18-2.** The P1 region of the parental virus and escape mutants was amplified and sequenced. Only the segment containing the mutated site(s) is shown. Red boxes indicate the positions of the identified substitutions.

**Fig. O. Sanger sequencing chromatograms of parent virus and escape mutants selected by pO18-28.** The P1 region of the parental virus and escape mutants was amplified and sequenced. Only the segment containing the mutated site(s) is shown. Red boxes indicate the positions of the identified substitutions.

**Fig. P. Sanger sequencing chromatograms of parent virus and escape mutants selected by pO18-29.** The P1 region of the parental virus and escape mutants was amplified and sequenced. Only the segment containing the mutated site(s) is shown. Red boxes indicate the positions of the identified substitutions.

**Fig. Q. Amplicon sequencing analysis of escape mutants selected by pO18-2, pO18-28, and pO18-29.** For each mutant, the figure lists the mutation position (nucleotide and corresponding amino acid), parental reference, mutant sequence, read depth, and allele frequency. The mutated nucleotide is highlighted in red.

**Fig. R. Characterization of VP3 mutant viruses and antigenic determinants. (A)** Sanger sequencing chromatograms of rescued VP3 mutant viruses derived from wild-type O/HN/CHA/93, including VP3 S70P, VP3 S70A, VP3 S70L, VP3 Q76R, and VP3 S70P/Q76R. **(B)** Plaque phenotypes of rescued VP3 mutant viruses described in (A). **(C)** Identification of key antigenic determinants recognized by three mAbs using O/HN/CHA/93 and the corresponding single-substitution mutant viruses. Neutralizing activity was evaluated by microneutralization assay. Virus neutralization titers (μg/mL) are shown using a color scale. **(D)** Sequence alignment of VP3 from representative O/Cathay strains. Residues at positions 70 and 76 are highlighted within blue boxes.

**Table A to G**

**Table A. Interface identification and interaction analysis of pO18-29 with FMDV O/HN/CHA/93 by PISA Program[1]**

| Interface | | | | Interaction | | | | |  |
| --- | --- | --- | --- | --- | --- | --- | --- | --- | --- |
| O/HN/CHA/93 | **pO18-29** | **BSA(Å^2^)^a^** | **Percentage^b^** | | **Type^c^** | **pO18-29^d^** | **Dist.(Å)** | **O/HN/CHA/93^e^** | |
| 3: HIS56 | H chain | 6.50 | \|\|\| | |  |  |  |  | |
| 3: PHE57 | H chain | 12.65 | \|\|\|\|\|\|\|\|\|\| | |  |  |  |  | |
| 3: GLU58 | H chain | 144.34 | \|\|\|\|\|\|\|\|\| | | H  S | H: TYR32[OH]  H:ARG102[NE] | 3.27  3.26 | 3:GLU58[OE2]  3:GLU58[OE2] | |
| 3: GLY59 | H chain | 6.70 | \|\| | |  |  |  |  | |
| 3: ASP60 | H chain | 14.40 | \|\| | |  |  |  |  | |
| 3: VAL64 | H chain | 8.87 | \|\|\|\|\|\|\| | |  |  |  |  | |
| 3: THR65 | H chain | 10.82 | \|\| | |  |  |  |  | |
| 3: LYS67 | H chain | 33.11 | \|\|\|\|\| | |  |  |  |  | |
| 3: ASP71 | H chain | 40.46 | \|\|\|\|\|\| | | H  S | H:ARG103[NH1]  H:ARG103[NH1] | 3.40  3.40 | 3:GLU58[OE2]  3:ASP71[OD1] | |
| 3: VAL73 | H chain | 29.50 | \|\|\|\|\|\|\|\|\|\| | |  |  |  |  | |
| 3: LEU74 | H chain | 12.97 | \|\|\|\|\|\|\|\|\| | |  |  |  |  | |
| 3: ALA75 | H chain | 7.20 | \|\|\|\|\|\|\|\|\| | |  |  |  |  | |
| 3: GLN76 | H chain | 68.50 | \|\|\|\|\|\|\|\|\|\| | | H  H  H | H:GLY107[N]  H:TYR110[OH]  H:ASN105[O] | 2.57  3.15  2.60 | 3:GLN76[O]  3:GLN76[OE1]  3:GLN76[N] | |
| 3: PHE77 | H chain | 6.53 | \|\|\|\| | |  |  |  |  | |
| 3: ASP78 | H chain | 7.65 | \|\| | |  |  |  |  | |
| 3: LYS84 | H chain | 79.67 | \|\|\|\|\|\| | | H | H:TRP27[O] | 2.63 | 3:LYS84[NZ] | |
| 3: HIS85 | H chain | 36.37 | \|\|\|\|\|\|\|\|\| | |  |  |  |  | |
| 3: GLU131 | H chain | 93.48 | \|\|\|\|\|\|\| | | H  H | H:ARG111[N]  H:TYR108[OH] | 3.73  3.30 | 3:GLU131[OE2]  3:GLU131[OE2] | |
| 3: PRO132 | H chain | 27.35 | \|\|\|\|\|\|\|\|\|\| | | H | H:TYR110[OH] | 2.36 | 3:PRO132[O] | |
| 3: LYS134 | H chain | 95.58 | \|\|\|\|\|\| | | H  S  S | H:ASP112[OD2]  H:ASP112[OD1]  H:ASP112[OD2] | 2.85  3.78  2.85 | 3:LYS134[NZ]  3:LYS134[NZ]  3:LYS134[NZ] | |
| 3: THR177 | H chain | 9.21 | \| | |  |  |  |  | |
| 3: TR183 | H chain | 32.00 | \|\|\|\|\|\| | |  |  |  |  | |
| 3: LYS67 | L chain | 3.59 | \| | |  |  |  |  | |
| 3: THR68 | L chain | 18.59 | \|\|\| | |  |  |  |  | |
| 3: ASP69 | L chain | 79.98 | \|\|\|\|\|\|\| | | H  H  S | L:ARG55[NH2]  L:ARG55[N]  L:ARG55[NH2] | 3.63  2.77  3.63 | 3:ASP69[OD1]  3:ASP69[OD2]  3:ASP69[OD1] | |
| 3: SER70 | L chain | 45.13 | \|\|\|\|\| | | H  H  H  H  H  H | L:ASN54[ND2]  L:ASP53[OD1]  L:ASN54[OD1]  L:GLY51[O]  L:ASP53[OD2]  L:ASN54[OD1] | 3.39  3.20  3.21  3.34  2.31  3.33 | 3:SER70[OG]  3:SER70[N]  3:SER70[N]  3:SER70[OG]  3:SER70[OG]  3:SER70[OG] | |
| 3: ASP71 | L chain | 37.40 | \|\|\|\|\| | | H | L:TYR50[OH] | 2.80 | 3:ASP71[OD2] | |
| 3: LYS134 | L chain | 24.37 | \|\| | | H | L:TYR33[OH] | 3.04 | 3:LYS134[NZ] | |
| 3: ASP195 | L chain | 8.08 | \| | |  |  |  |  | |
| 1: PRO196 | L chain | 0.33 | \| | |  |  |  |  | |
| 1: SER197 | L chain | 3.71 | \| | |  |  |  |  | |

a BSA: Buried Surface Area; b ||||: Buried area percentage, one bar per 10%. c H, Hydrogen bond; S, Salt bridge. d: Interactive atoms of amino acid side chain of pO18-29. e: Interactive atoms of amino acid side chain of O/HN/CHA/93.

**Table B. Interface identification and interaction analysis of pO18-29 with FMDV O/18074 by PISA Program[1]**

| Interface | | | | Interaction | | | | |  |
| --- | --- | --- | --- | --- | --- | --- | --- | --- | --- |
| O/18074 | **pO18-29** | **BSA(Å^2^)^a^** | **Percentage^b^** | | **Type^c^** | **pO18-29^d^** | **Dist.(Å)** | **O/18074^e^** | |
| 3: ARG56 | H chain | 12.96 | \|\|\| | |  |  |  |  | |
| 3: PHE57 | H chain | 7.78 | \|\|\|\|\|\|\|\|\| | |  |  |  |  | |
| 3: ASP58 | H chain | 96.47 | \|\|\|\|\|\|\|\|\| | | H  H  H  H  S  S | H:ARG102[NH2]  H:ARG102[NH2]  H:ASN105[N]  H:ASN105[OD1]  H:ARG102[NH2]  H:ARG102[NH2] | 3.84  3.63  3.62  3.04  3.84  3.63 | 3:ASP58[OD1]  3:ASP58[OD2]  3:ASP58[OD2]  3:ASP58[N]  3:ASP58[OD1]  3:ASP58[OD2] | |
| 3: SER59 | H chain | 17.61 | \|\| | |  |  |  |  | |
| 3: ASP60 | H chain | 4.54 | \| | |  |  |  |  | |
| 3: TYR63 | H chain | 0.12 | \| | |  |  |  |  | |
| 3: VAL64 | H chain | 7.70 | \|\|\|\|\|\| | |  |  |  |  | |
| 3: LYS67 | H chain | 22.09 | \|\|\|\| | |  |  |  |  | |
| 3: ASP71 | H chain | 42.53 | \|\|\|\|\|\| | | S  S | H:ARG103[NH1]  H:ARG103[NH1] | 3.39  3.61 | 3:ASP71[OD2]  3:ASP71[OD1] | |
| 3: LYS73 | H chain | 36.61 | \|\|\|\|\|\|\|\|\|\| | | H  H | H:TYR110[OH]  H:TYR116[OH] | 3.03  3.38 | 3:LYS73[NZ]  3:LYS73[NZ] | |
| 3: LEU74 | H chain | 20.26 | \|\|\|\|\|\|\|\|\| | | H | H:ASN105[ND2] | 3.87 | 3:LEU74[O] | |
| 3: VAL75 | H chain | 9.87 | \|\|\|\|\|\|\|\|\| | |  |  |  |  | |
| 3: GLN76 | H chain | 66.85 | \|\|\|\|\|\|\|\|\|\| | | H  H  H  H  H | H:GLY107[N]  H:GLY107[N]  H:TYR108[N]  H:ASN105[O]  H:TYR110[OH] | 3.33  3.42  3.04  2.83  2.75 | 3:GLN76[O]  3:GLN76[OE1]  3:GLN76[OE1]  3:GLN76[N]  3:GLN76[NE2] | |
| 3: PHE77 | H chain | 1.17 | \|\| | |  |  |  |  | |
| 3: ASP78 | H chain | 5.23 | \| | |  |  |  |  | |
| 3: LYS84 | H chain | 51.59 | \|\|\|\| | | H | H:TRP27[O] | 2.85 | 3:LYS84[NZ] | |
| 3: HIS85 | H chain | 22.09 | \|\|\|\|\|\| | | H | H:ASN105[OD1] | 3.85 | 3:HIS85[NE2] | |
| 3: MET130 | H chain | 0.59 | \| | |  |  |  |  | |
| 3: GLU131 | H chain | 106.27 | \|\|\|\|\|\|\|\| | | H  H  H  S  S | H:TYR108[OH]  H:ARG111[N]  H:ARG111[NH1]  H:ARG111[NH1]  H:ARG111[NH1] | 3.84  3.28  2.99  2.99  3.83 | 3:GLU131[OE2]  3:GLU131[OE2]  3:GLU131[OE1]  3:GLU131[OE1]  3:GLU131[OE2] | |
| 3: PRO132 | H chain | 26.26 | \|\|\|\|\|\|\|\|\| | | H | H:TYR110[OH] | 2.91 | 3:PRO132[O] | |
| 3: THR134 | H chain | 46.94 | \|\|\|\|\| | |  |  |  |  | |
| 3: VAL174 | H chain | 0.34 | \| | |  |  |  |  | |
| 3: THR177 | H chain | 54.53 | \|\|\|\|\| | |  |  |  |  | |
| 3: THR178 | H chain | 42.78 | \|\|\|\|\| | |  |  |  |  | |
| 3: TRP183 | H chain | 17.76 | \|\|\|\|\|\|\| | |  |  |  |  | |
| 3: LYS67 | L chain | 6.25 | \| | |  |  |  |  | |
| 3: THR68 | L chain | 25.47 | \|\|\|\| | | H  H | L:ARG55[NH2]  L:ARG61[NH1] | 3.70  3.72 | 3:THR68[OG1]  3:THR68[OG1] | |
| 3: ASP69 | L chain | 76.33 | \|\|\|\|\|\|\| | | H  S | L:ARG55[N]  L:ARG55[NH2] | 3.53  3.99 | 3:ASP69[OD1]  3:ASP69[OD2] | |
| 3: SER70 | L chain | 44.88 | \|\|\|\|\| | | H  H  H  H  H  H | L:ASN54[ND2]  L:ASP53[OD1]  L:ASP53[OD2]  L:ASN54[OD1]  L:ASP53[OD1]  L:GLY51[O] | 3.24  3.12  3.02  3.24  2.43  3.09 | 3:SER70[OG]  3:SER70[N]  3:SER70[N]  3:SER70[N]  3:SER70[OG]  3:SER70[OG] | |
| 3: ASP71 | L chain | 40.67 | \|\|\|\|\| | | H | L:TYR50[OH] | 3.15 | 3:ASP71[OD2] | |
| 3: THR134 | L chain | 4.90 | \| | |  |  |  |  | |
| 3: ASP195 | L chain | 1.18 | \| | |  |  |  |  | |
| 1: PRO196 | H chain | 0.33 | \| | |  |  |  |  | |
| 1: SER197 | H chain | 3.71 | \| | |  |  |  |  | |
| 1: SER197 | L chain | 56.70 | \|\|\|\|\| | |  |  |  |  | |
| 1: THR198 | L chain | 54.93 | \|\|\|\|\|\|\| | |  |  |  |  | |
| 1: ALA199 | L chain | 50.54 | \|\|\|\|\| | | H | H:ASP28[OD2]  H:ASN31[OD1] | 3.49  3.86 | 1:ALA199[N]  1:ALA199[N] | |
| 1: LYS202 | L chain | 2.80 | \| | |  |  |  |  | |

a BSA: Buried Surface Area; b ||||: Buried area percentage, one bar per 10%. c H, Hydrogen bond; S, Salt bridge. d: Interactive atoms of amino acid side chain of pO18-29. e: Interactive atoms of amino acid side chain of O/18074.

**Table C. FMDV O/HN/CHA/93 with pO18-29 interaction residues.**

| **Domain** | **Residue** | **Distance(Å)** | **pO18-29** | **CDR** |
| --- | --- | --- | --- | --- |
| **VP3 B-B knob** | H56(NE2) | 3.3 | W27(NE1) | HCDR1 |
|  | F57(CA) | 3.7 | W27(CZ2) | HCDR1 |
|  | E58(OE2) | 3.3 | R102(NE) | HCDR3 |
|  | E58(OE2) | 3.3 | Y32(OH) | HCDR1 |
| **VP3 βB** | V64(CG1) | 3.4 | N105(OD1) | HCDR3 |
| **VP3 B-C loop** | K67(NZ) | 3.6 | K104(CB) | HCDR3 |
|  | D69(OD2) | 2.8 | R55(N) | L-FR3 |
|  | D69(OD1) | 3.6 | R55(NH2) | L-FR3 |
|  | S70(OG) | 3.3 | G51(O) | LDCR2 |
|  | S70(OG) | 2.3 | D53(OD2) | LCDR2 |
|  | S70(N) | 3.2 | D53(OD1) | LCDR2 |
|  | S70(OG) | 3.4 | N54(ND2) | L-FR2 |
|  | S70(OG) | 3.3 | N54(OD1) | L-FR3 |
|  | S70(N) | 3.2 | N54(OD1) | L-FR3 |
|  | D71(OD1) | 3.4 | R103(NH1) | HCDR3 |
|  | D71(OD2) | 2.8 | Y50(OH) | L-FR2 |
|  | D71(N) | 2.6 | N54(OD1) | L-FR3 |
| **VP3 βC** | V73(CG1) | 3.7 | V106(CG2) | HCDR3 |
|  | L74(O) | 3.3 | N105(CB) | HCDR3 |
|  | A75(CA) | 3.4 | N105(O) | HCDR3 |
|  | Q76(N) | 2.6 | N105(O) | HCDR3 |
|  | Q76(O) | 2.6 | G107(N) | HCDR3 |
|  | Q76(OE1) | 3.2 | Y110(OH) | HCDR3 |
| **VP3 C-D loop** | K84(NZ) | 2.6 | W27(O) | HCDR1 |
|  | H85(NE2) | 3.2 | W27(CZ3) | HCDR1 |
| **VP3 E-F loop** | E131(OE2) | 3.3 | Y108(OH) | HCDR3 |
|  | E131(OE2) | 3.7 | R111(N) | HCDR3 |
|  | P132(O) | 2.4 | Y110(OH) | HCDR3 |
|  | K134(NZ) | 3.8 | D112(OD1) | HCDR3 |
|  | K134(NZ) | 2.9 | D112(OD2) | HCDR3 |
|  | K134(NZ) | 3 | Y33(OH) | LCDR1 |
| **VP3 βH** | W183(NE1) | 3.4 | Y108(CD1) | HCDR3 |

The interaction residues were computed using the CCP4[2] hydrogen bond distance cutoff of 4.0 Å and the salt-bridge distance cutoff of 4.0 Å. The blue font refers to a hydrogen bond. The orange font refers to salt bridge.

**Table D. FMDV O/18074 with pO18-29 interaction residues.**

| **Domain** | **Residue** | **Distance(Å)** | **pO18-29** | **CDR** |
| --- | --- | --- | --- | --- |
| **VP3 B-B knob** | R56(NH1) | 2.9 | W27(CZ2) | HCDR1 |
|  | F57(CB) | 3.7 | N105(ND2) | HCDR3 |
|  | D58(OD1) | 3.8 | R103(NH2) | HCDR3 |
|  | D58(OD2) | 3.6 | R102(NH2) | HCDR3 |
|  | D58(OD2) | 3.6 | N105(N) | HCDR3 |
|  | D58(N) | 3 | N105(OD1) | HCDR3 |
|  | S59(N) | 3.1 | W27(CH2) | HCDR1 |
|  | D60(N) | 3.7 | W27(CH2) | HCDR1 |
| **VP3 βB** | V64(CG1) | 3.8 | N105(ND2) | HCDR3 |
| **VP3 B-C loop** | T68(OG1) | 3.7 | R55(NH2) | L-FR3 |
|  | T68(OG1) | 3.7 | R61(NH1) | L-FR3 |
|  | D69(OD1) | 3.5 | R55(N) | L-FR3 |
|  | D69(OD2) | 4 | R55(NH2) | L-FR3 |
|  | S70(OG) | 3.1 | G51(O) | LCDR2 |
|  | S70(OG) | 2.4 | D53(OD1) | LCDR2 |
|  | S70(N) | 3.1 | D53(OD1) | LCDR2 |
|  | S70(N) | 3 | D53(OD2) | LCDR2 |
|  | S70(OG) | 3.2 | N54(ND2) | L-FR3 |
|  | S70(N) | 3.2 | N54(OD1) | L-FR3 |
|  | D71(OD2) | 3.2 | Y50(OH) | L-FR2 |
|  | D71(N) | 2.8 | N54(OD1) | L-FR3 |
|  | D71(OD1) | 3.6 | R103(NH1) | HCDR3 |
|  | D71(OD2) | 3.4 | R103(NH1) | HCDR3 |
| **VP3 βC** | K73(NZ) | 3 | Y110(OH) | HCDR3 |
|  | K73(NZ) | 3.4 | Y116(OH) | HCDR3 |
|  | L74(O) | 3.9 | N105(ND2) | HCDR3 |
|  | V75(CA) | 3.5 | N105(O) | HCDR3 |
|  | Q76(N) | 2.8 | N105(O) | HCDR3 |
|  | Q76(O) | 3.3 | G107(N) | HCDR3 |
|  | Q76(OE1) | 3.4 | G107(N) | HCDR3 |
|  | Q76(OE1) | 3 | Y108(N) | HCDR3 |
|  | Q76(NE2) | 2.7 | Y110(OH) | HCDR3 |
| **VP3 C-D loop** | K84(NZ) | 2.9 | W27(O) | HCDR1 |
|  | H85(NE2) | 3.9 | N105(OD1) | HCDR3 |
| **VP3 E-F loop** | E131(OE2) | 3.8 | Y108(OH) | HCDR3 |
|  | E131(OE2) | 3.3 | R111(N) | HCDR3 |
|  | E131(OE1) | 3 | R111(NH1) | HCDR3 |
|  | E131(OE2) | 3.8 | R111(NH1) | HCDR3 |
|  | P132(O) | 2.9 | Y110(OH) | HCDR3 |
|  | T134(CG2) | 3.6 | Y110(CZ) | HCDR3 |
|  | T134(CG2) | 3.8 | Y116(CE1) | HCDR3 |
| **VP3 G-H loop** | T177(CB) | 3.5 | V55(N) | HCDR2 |
|  | T178(CG2) | 3.8 | V55(CB) | HCDR2 |
| **VP3 βH** | W183(CZ2) | 3.5 | Y108(CE2) | HCDR3 |
| **VP1 C-terminal** | S197(O) | 2.6 | W27(O) | HCDR1 |
|  | T198(OG1) | 3.3 | P30(CG) | HCDR1 |
|  | A199(N) | 3.5 | D28(OD2) | HCDR1 |
|  | A199(N) | 3.9 | N31(OD1) | HCDR1 |

The interaction residues were computed using the CCP[2] hydrogen bond distance cutoff of 4.0 Å and the salt-bridge distance cutoff of 4.0 Å. The blue font refers to a hydrogen bond. The orange font refers to salt bridge.

**Table E. Interactions and total stabilizing energy of pO18-29 with FMDV O/HN/CHA/93 or O/18074.**

|  | OHN-pO18-29 | O18-pO18-29 |
| --- | --- | --- |
| Number of interface residues | 98 | 111 |
| Normalized Energy per residue | -4.82 kJ/mol | -4.16 kJ/mol |
| No. of Short Contacts | 16 | 17 |
| No. of Hydrophobic Interactions | 1 | 0 |
| No. of van der Waals Pairs | 9918 | 10691 |
| No. of Salt Bridges | 6 | 6 |
| No. of Potential Favourable Electrostatic Interactions | 12 | 10 |
| No. of Potential Unfavourable Electrostatic Interactions | 9 | 8 |
| Hydrogen Bond Energy | -108.98 kJ/mol | -82.14 kJ/mol |
| Electrostatic Energy | -81.18 kJ/mol | -81.46 kJ/mol |
| Van der Waals Energy | -281.77 kJ/mol | -297.88 kJ/mol |
| Total Stabilizing Energy | -471.94 kJ/mol | -461.47 kJ/mol |

**Table F. FMDV strains used in this study.**

| Serotype | Topotype | Strain | Isolation year | Origin / Host |
| --- | --- | --- | --- | --- |
| O | Cathay | O/HN/CHA/93 | 1993 | China / pig |
| O | Cathay | O/SCGH/CHA/2016 | 2016 | China / pig |
| O | Cathay | O/18074 | 2018 | China / pig |
| O | Cathay | O/GD/2021 | 2021 | China / pig |
| O | Cathay | O/GX/2022 | 2022 | China / pig |
| O | ME-SA (PanAsia) | O/Tibet/99 | 1999 | China /cattle |
| O | ME-SA (Ind2001) | O/GZ1/2025 | 2025 | China /cattle |
| O | ME-SA (Ind2001) | O/XJ/CHA/2017 | 2017 | China /cattle |
| O | SEA (Mya98) | O/GSLX/2010 | 2010 | China / pig |
| O | SEA (Mya98) | O/CHN/Mya98/33-p | 2010 | China /cattle |
| O | SEA (Mya98) | O/HNNY/2022 | 2022 | China / pig |
| A | ASIA (A22) | A/AF72 | 1972 | China / cattle |
| A | ASIA (Sea97) | A/WH/CHA/09 | 2009 | China / cattle |
| A | ASIA (Sea97) | A/GDMM/2013 | 2013 | China / pig |

**Table G. Cryo-EM data collection and refinement statistics.**

|  | FMDV-OHN-pO18-29 | FMDV-O18-pO18-29 |
| --- | --- | --- |
| Data collection and processing |  |  |
| Magnification | 105,000 | 105,000 |
| Voltage (kV) | 300 | 300 |
| Electron exposure (e^–^/Å^2^) | 60 | 60 |
| Defocus range (μm) | -2.2 to -1.0 | -2.2 to -1.0 |
| Pixel size (Å) | 0.83 | 0.83 |
| Symmetry imposed | I1 | I1 |
| Final particle images (no.) | 59,569 | 25,588 |
| Map resolution (Å) | 2.24 | 2.34 |
| Map sharpening B factor (Å^2^) | -79.8 | -77.7 |
| Model Building and validation |  |  |
| Initial model used (PDB code) | 8Y0Q | 8Y0Q |
| MolProbity score | 1.55 | 1.83 |
| Clash score | 6.02 | 8.02 |
| Rotamer outliers (%) | 0.00 | 0.00 |
| R.M.S.D. of bond lengths (Å) | 0.003 | 0.005 |
| R.M.S.D. of angles (∘) | 0.576 | 0.671 |
| Favored (%) | 96.54 | 94.18 |
| Allowed (%) | 3.34 | 5.82 |
| Outliers (%) | 0.12 | 0.00 |

# **References**

1.	Krissinel, E. and K. Henrick, *Inference of macromolecular assemblies from crystalline state.* J Mol Biol, 2007. **372**(3): p. 774-97.

2.	Winn, M.D., et al., *Overview of the CCP4 suite and current developments.* Acta Crystallogr D Biol Crystallogr, 2011. **67**(Pt 4): p. 235-42.
