# Supplementary material for: Structural basis of antigenic drift in FMDV O/Cathay topotype variants revealed by porcine-derived single B-cell neutralizing antibodies
Source: PLoS Pathog. 2026 Oct 15;22(10):e1014650. doi: 10.1371/journal.ppat.1014650 (PMC13671523; doi:10.1371/journal.ppat.1014650)
Supplement: Supplementary file 2 [file ppat.1014650.s002.docx]

Dear editors and reviewers:

Thank you for your letter and the reviewers' comments on our manuscript titled "Structural basis of antigenic drift in FMDV O/Cathay topotype variants revealed by porcine-derived single B-cell neutralizing antibodies". These comments have been highly valuable and have greatly contributed to the revision and improvement of our paper. They have also provided important guidance for our further research.

We have also carefully addressed the editorial requests regarding data and formatting. All tables have been removed as separate files and are now embedded as editable, cell‑based objects within the manuscript, placed immediately after their first citation. A full list of legends for the Supporting Information files has also been added after the reference list. The cryo‑EM maps and coordinates (EMD‑69003/PDB 23IT and EMD‑69004/PDB 23IU) are currently being released and will be publicly accessible on September 9, 2026. We confirm that Fig. 2A was created using BioRender.com under an active publication license. We have reviewed the amended Competing Interest statement and confirm that it is correct.

We have thoroughly reviewed all the comments and made corresponding corrections accordingly. All figures have been carefully examined and adjusted using PLOS’s NAAS tool. Revised content in the supplementary information has been highlighted in yellow. The main corrections in the paper, along with our point‑by‑point responses to the editors' and reviewers' comments, are outlined below.

Best wishes,

Kun Li

Reviewer's Responses to Questions

Part I - Summary
Please use this section to discuss strengths/weaknesses of study, novelty/significance, general execution and scholarship.

Reviewer #1: The manuscript presents a well-executed study on antigenic drift in FMDV topotype variants using porcine-derived single B cell neutralizing antibodies. The study is methodologically solid, combining reverse genetics, scFv production, and cryo-electron microscopy to characterize antibody–virus interactions. The work is relevant for understanding antigenic variability and its implications for immune escape and vaccine design in swine populations. Overall, the results are convincing, and the main novelty lies in the functional and structural validation of antigenic determinants in infectious virus systems.

Response:

Thanks for the comments.

Reviewer #2: Li et al., have used a panel of seven porcine-derived neutralizing monoclonal antibodies (pnAbs) to identify antigenic regions responsible for neutralization escape from porcine immune sera in recent FMDV O/Cathay variants. A charge modification at +2 residue (Q→H/R) downstream the conserved RGD receptor-binding motif on VP1 (within a region well characterized as a relevant B cell antigenic site), modulates sensitivity to different pnAbs and vaccine-induced sera. In addition, cryo-EM structures of one of the pnAbs (pO18-29) in complex with the ancient O/HN/CHA/93 strain (the strain used as a reference in this study) and the recent variant O/18074 revealed a novel conformation-dependent, VP3-centered antigen determinant. Substitutions leading to charge modifications at positions 73 and 134 on VP3 disrupt hydrophobic contacts, hydrogen bonds, and salt bridges, leading the virus to escape different antibodies. Interestingly, these two VP3 residues act cooperatively to regulate antibody recognition.
The authors conclude that the results contribute to elucidate the structural basis of neutralizing evasion in O/Cathay and support the rational design of next-generation broad-spectrum FMDV vaccine against emerging O/Cathay variants.
General comments
The topic of this manuscript is interesting as O/Cathay topotype has accumulated mutations over time associated with antigenic drift that can become a real problem in terms of vaccine effectiveness in different Asian regions. In general, this manuscript is well written and describes a comprehensive and, in my opinion, well conducted original work. Porcine-derived neutralizing mAbs are also interesting and suited tools for the analysis of the antigenic structures recognized by pig sera.
Nevertheless, there are issues regarding data and manuscript presentation as well as discussion that have to be addressed. In this line, the interesting statement at the end of the Abstract “Together, our findings elucidate ….. and provide a molecular framework for rational design of next-generation broad-spectrum FMDV vaccine …… “deserves further attention. The authors can discuss in more detail how these kinds of results can be translated into vaccine optimization and matching, as well as their potential impact on conventional as well as subunit vaccines.
In addition, a comparison of the genetic distances among the type O viruses analyzed, such as a P1 phylogenetic tree, will be valuable to determine correlations between genetic drift (nucleotide substitutions) and the antigenic changes among the O/Cathay variants. The inclusion of such comparison will improve the analyses of the data presented.

Response:

Thanks very much for the advice. These comments are valuable and contributed great for improvement of the manuscript. In response to the suggestion regarding vaccine implications, we have added the following statement at the end of Discussion section: “Specifically, the identification of VP1 residue 149 (RGD+2) and VP3 residues 73 and 134 as critical determinants of neutralization escape provides clear targets for monitoring antigenic drift in the field, potentially guiding strain selection for conventional vaccine updates. In addition, the structural characterization of these epitopes may facilitate the design of subunit immunogens that help broaden vaccine‑induced immunity.”

Furthermore, we have constructed a P1-based phylogenetic tree incorporating O/Cathay isolates from different years, which shows that the 2016–2022 variants form a distinct clade (Supplementary Fig. S2A). This correlates directly with their rank order of neutralization resistance observed in Fig. 1 (O/GX/2022 > O/GD/2021 > O/18074 > O/SCGH/CHA/2016), providing evidence that genetic drift in P1 is associated with progressive antigenic change within the Cathay topotype. This analysis has been added to the revised manuscript (Results, lines 130-134).

Reviewer #3:  It has been reported that newly emerging FMDV O/Cathay strains circulating in China can escape neutralization by sera from vaccinated pigs due to the accumulation of mutations that reduce antibody-mediated neutralization and compromise vaccine efficacy. In this manuscript, the authors investigate recently emerged O/Cathay variants that exhibit this immune escape phenotype. The main strength and novelty of the study lies in the structural characterization of the molecular determinants underlying antigenic divergence through the combined use of cryo-electron microscopy and porcine neutralizing monoclonal antibodies.

The authors identify two key antigenic regions involved in immune escape: one located adjacent to the RGD motif in VP1 and a second, previously unrecognized, region in VP3. They demonstrate that two point mutations affecting VP3 disrupt critical molecular interactions and act cooperatively to confer resistance to antibody-mediated neutralization.

The methodology used is appropriate for the study’s objectives and is described in sufficient detail. However, the use of unpaired t-tests with Holm–Šidák correction is not statistically appropriate for this experimental design, which compares vaccine-induced neutralizing antibody titers against the homologous virus and four viral variants. A more appropriate approach would be a one-way ANOVA followed by Dunnett’s multiple-comparison test if the objective is to compare each viral variant exclusively with the homologous virus, or Tukey’s multiple-comparison test if all pairwise comparisons among variants are intended.

Recent outbreaks of foot-and-mouth disease in China have also been caused by two additional serotype O topotypes, Middle East–South Asia (ME-SA) and Southeast Asia (SEA). Is it known whether these topotypes, like Cathay, also exhibit escape from vaccine-induced neutralization? If so, are these escape phenotypes associated with the same mutations and structural alterations identified in the present study? Otherwise, to what extent can the conclusions of this manuscript be generalized to other serotype O topotypes?

Overall, the study is scientifically relevant and provides valuable structural insights into the mechanisms by which emerging FMDV variants evade vaccine-induced immune responses. These findings improve our understanding of the antigenic evolution of the O/Cathay topotype and provide a rational framework for the design of next-generation vaccines with broader cross-protective efficacy against antigenically divergent strains.

Response:

Thanks for the valuable comments. We have revised the statistical analysis as suggested. The unpaired t-tests have been replaced with one-way ANOVA followed by Dunnett's multiple-comparison test in Fig. 1, Fig. S1 and Fig. S11.

In addition, to assess whether ME-SA and SEA topotypes escape vaccine-induced neutralization, we performed additional cross-neutralization assays against the recently emerged ME-SA (O/GZ1/2025) and SEA (O/HNNY/2022) strains using both polyclonal porcine sera and the seven porcine mAbs. O/HNNY/2022 showed marginal escape, substantially weaker than the O/Cathay variants, while O/GZ1/2025 showed no significant escape (Fig. S1). Both strains were efficiently neutralized by all seven mAbs (Fig. 2F). These findings indicate that ME-SA and SEA strains do not display the pronounced escape phenotype characteristic of the O/Cathay variants. These findings suggest that the escape mechanisms identified here are specific to the O/Cathay topotype and are not shared by ME‑SA or SEA strains. This additional data has been incorporated into the revised manuscript (Results, lines 120-129 and 175-179).

Part II – Major Issues: Key Experiments Required for Acceptance
Please use this section to detail the key new experiments or modifications of existing experiments that should be absolutely required to validate study conclusions.

Generally, there should be no more than 3 such required experiments or major modifications for a "Major Revision" recommendation. If more than 3 experiments are necessary to validate the study conclusions, then you are encouraged to recommend "Reject".

Reviewer #1: There are no major experimental issues that would be required to support the conclusions of the study. The work is overall well designed and the experimental approaches are appropriate for the objectives of the manuscript.

Response:

Thanks for the comments.

Reviewer #2:

Question 1

The presence of the mutations selected in the neutralization-escape mutants (Table 1), as well as those in the site-directed mutants (Fig. S1A), should be confirmed in the viral stocks from which the different analyses were conducted.

Response:

We agree with the reviewer that the mutations in the viral stocks should be confirmed. We have therefore added Sanger sequencing chromatograms for the neutralization-escape mutants and site-directed mutants (Figs. S4-S6, S8A, S10A, S12A, S13B, S14-S16, and S18A), confirming the presence of the intended mutations. In addition, to quantify the mutation frequencies in the viral stocks, the parental virus and seven representative escape mutants (one per pnAb) were subjected to amplicon sequencing. The results are presented in Figs. S7, S12B, and S17. The amplicon sequencing results were largely consistent with the Sanger sequencing data, with one notable exception: a pO18‑2 escape mutant carrying Q76R also harbored additional mutations at VP3 residue 134 (K→E, 0.333; K→T, 0.414). These data have been incorporated into the Results section.

Reviewer #3: (No Response)

Part III – Minor Issues: Editorial and Data Presentation Modifications
Please use this section for editorial suggestions as well as relatively minor modifications of existing data that would enhance clarity.

Reviewer #1:

Question 2

Only minor improvements are suggested to further improve clarity. In particular, the resolution and labeling of some figures could be enhanced for better readability. In addition, a minor error in the abstract regarding antibody naming should be corrected,

These are minor points and do not affect the overall validity or strength of the study

Response：Thanks for these constructive suggestions. We have carefully reviewed the resolution and labeling of all figures and made necessary adjustments to improve clarity. The minor error in the Abstract regarding antibody naming has also been corrected. In addition, we have thoroughly revised the writing throughout the manuscript.

Reviewer #2: Specific Comments

Question 3
The type O viruses used in this study in neutralization assays, as well as those employed in the vaccines for pig immunization (as in Fig. 1), should be clearly indicated. In particular, the date of isolation of O/18074 isolate should be provided. As this information is relevant for the data discussion, I recommend its inclusion as a new Table.
Response: Thanks for the advice. We have now compiled all strains information into a new supplementary table (Table S6), which includes the serotype, topotype, strain name, isolation year, and origin/host for each virus used in the neutralization assays and vaccine preparation. The isolation date of O/18074 (2018) has been included in the Table S6.

Question 4
The number of passages given to the viral populations, and the stability of the mutations selected in the neutralization-scape mutants (Table 1), as well as those of the site-directed mutants (Fig. S1A) should be clearly indicated and discussed.
Response: Thanks for this important question. In the Materials and Methods section, we have added the number of passages for both the escape mutants and the site‑directed mutants. For the escape mutants: “For each antibody, four escape mutants (A-D) were selected using an initial mAb concentration of 10 μg/ml, and four additional mutants (E-H) were selected using 20 μg/ml. The A–D mutants were serially passaged for seven rounds up to 640 μg/ml, whereas the E–H mutants were passaged for six rounds to reach the same final concentration.” For the site‑directed mutants: “At 72 h post‑transfection, the culture supernatants and cells were harvested and passaged to the fifth passage on BHK‑21 cells.” Regarding mutation stability, Sanger sequencing and amplicon sequencing of the viral stocks confirmed the presence of the intended mutations and their high‑frequency retention after passaging (Figs. S4-8, S10, 12-18). These data have been incorporated into the revised manuscript.

Question 5

Likewise, the rationale for selecting the pnmAb used for Cryo-EM analyses (pO18-29) should be discussed

Response: Thanks for this question. The rationale for selecting pO18‑29 for cryo‑EM analysis has been added to the Results section: “we selected pO18‑29 for cryo‑EM analysis, as it exhibited potent neutralization against the ancient O/HN/CHA/93 strain but not against the recent variants (2016–2018).” This statement has been incorporated into the revised manuscript (lines 231-233).

Question 6
Line 131. (Fig. 2B). Why the results with O/18074 in IFA are not shown?
Response: Thanks for this question. The IFA results for the O/HN/CHA/93 strain were shown in Fig. 2B to illustrate the differential binding intensities among the seven mAbs against the ancient O/Cathay strain. As the binding signals of all mAbs to the variant O/18074 were uniformly weak, these results have been placed in the supplementary materials (Fig. S3). A corresponding description has been added to the main text: "All mAbs exhibited only weak binding to O/18074 in IFA (Fig. S3)". This newly added data is now included in the revised manuscript (lines 151).

Question 7
Line 133. The reason why the BLI kinetic analyses revealed that all pmAbs bound O/HN/CHA/93 with greater affinity than the recent variant O/18074 is not evident from what is commented in this section of the Results. This should be revised and the viruses used for the immunization of the pigs for the production of FMDV-specific porcine B-cell repertoires indicated (this reviewer has not been able to access reference 40).
Response: In the revised manuscript, we have clarified the immunization protocol used for BCR repertoire generation and have cited the relevant literature: the pig was primed and boosted with O/HN/CHA/93 prior to the third immunization with O/18074 and other heterologous strains (Results, lines 140-142). We have also added an explanation that the preferential binding of O/18074‑derived mAbs to the ancestral O/HN/CHA/93 strain is consistent with original antigenic sin (Results, lines 159-161).

Other points

Question 8
Abstract, line 22: include “that” in the sentence “Herein, we observed (that) the new emerging …. … “
Response: Thanks for this correction. We have added "that" in the Abstract as per your suggestion.

Question 9
Abstract, line 32: delete “that” in the sentence: “Moreover, residues 73 and 134 that synergistically regulate pnAbs (pO18-2, pO18-18, pO18-29 and pO18-29) recognition”.
Response: Thanks, we have deleted the unnecessary "that" in the Abstract as suggested.

Reviewer #3:

Question 10

Figures 1A–D would benefit from using the same y-axis scale (up to 4 log), which would facilitate direct comparison across panels.
Response: Thanks for the advice. We have now aligned the y-axis scales across all panels in Fig. 1A-D, using a common scale up to 4 log10, to facilitate direct comparison across the panels.

Question 11

Lines 124–125; Figures 2C–E. The manuscript states that the monoclonal antibodies (mAbs) were derived from the previously established O/18074-specific porcine B-cell receptor repertoire (“55 mAb clones from the previously established O/18074-specific porcine BCR repertoire”). How do the authors explain that several mAbs exhibit higher binding affinity for the ancestral virus O/HN/CHA/93 than for the homologous virus O/18074 (Figures 2C–E)? In this context, Figure 2B should also include the binding reactivity of the mAbs against O/18074, rather than only against the ancestral virus. Alternatively, could there be an error in the manuscript, with the mAbs actually being generated from O/HN/CHA/93?
Response: We confirm that the mAbs were indeed derived from the O/18074-specific BCR repertoire, as stated in the manuscript, and there is no error in this regard. In the revised manuscript, we have clarified the immunization protocol used for BCR repertoire generation: the pig was primed and boosted with O/HN/CHA/93 prior to the third immunization with O/18074 and other heterologous strains. The preferential binding of O/18074-derived mAbs to the ancestral O/HN/CHA/93 strain over the homologous variant is consistent with original antigenic sin, as the immune response was imprinted by the initial exposure to O/HN/CHA/93. Regarding Figure 2B, the IFA results for O/HN/CHA/93 were shown in the main figure to illustrate the differential binding intensities among the seven mAbs. As the binding signals of all mAbs to O/18074 were uniformly weak, we have now included these results as Supplementary Fig. S3 in the revised manuscript. We have also added a corresponding description in the main text: "All mAbs exhibited only weak binding to O/18074 in IFA (Fig. S3)." These revisions have been incorporated into the revised manuscript (Results, lines 140-142, line 151, and 159-161; Fig. S3).

Question 12

Figure 5. The figure would be clearer and facilitate comparison if the expanded views of the antibody–antigen interfaces were reorganized into two columns: one for O/HN/CHA/93 and the other for O/18074. Within each column, the panels should be arranged in the order E–F loop, B–C loop, and BB-knob. This layout would allow direct side-by-side comparison of the corresponding structural elements in both viruses. Using the current panel nomenclature, the proposed order would be: A, E, C, G, B, F, D, and H.
Response: Thanks for the advice. We have reorganized Figure 5 into two columns as recommended (left: O/HN/CHA/93; right: O/18074), with panels ordered as E-F loop, B-C loop, and BB-knob (A, E, C, G, B, F, D, H). This layout facilitates direct side-by-side comparison. The panel nomenclature has been updated accordingly in the revised figure and legend.

Question 13
Line 259. The statement “All recombinant viruses were successfully rescued (Fig. S2A–B)” appears to cite the wrong figure, as Figure S2 corresponds to the cryo-EM analysis rather than the rescued viruses. The correct citation should be Figures S1A–C.
Response: Thanks to the reviewer for catching this error. Due to the reorganization of supplementary figures during revision, the figure numbering has changed. We have corrected the citation accordingly, and the statement now reads: “All recombinant viruses were successfully rescued and confirmed by Sanger sequencing and plaque assays (Fig. S10)” (Lines 338-339). All related citations have been updated throughout the manuscript to reflect the current numbering.

Question 14
Figure S3. The y-axis label should be revised from “Dilution (lg)” to “Serum neutralization titers (log).”
Response: We have revised the y‑axis label from “Dilution (lg)” to “Serum neutralization titers (log)” as recommended. Please note that due to the renumbering of supplementary figures during revision, the figure previously cited as Fig. S3 is now designated as Fig. S11 in the revised manuscript. All related citations have been updated accordingly.
