# Supplementary material for: Structural basis of antigenic drift in FMDV O/Cathay topotype variants revealed by porcine-derived single B-cell neutralizing antibodies
Source: PLoS Pathog. 2026 Oct 15;22(10):e1014650. doi: 10.1371/journal.ppat.1014650 (PMC13671523; doi:10.1371/journal.ppat.1014650)
Supplement: Peer review history

# Peer review: Decision Letter 0

**DOI:** 10.1371/journal.ppat.1014650.r001

**Authors:** Sonja M. Best, Margarita Saiz

2 Jul 2026

Structural basis of antigenic drift in FMDV O/Cathay topotype variants revealed by porcine-derived single B-cell neutralizing antibodies

PLOS Pathogens

Dear Dr. Li,

Thank you for submitting your manuscript to PLOS Pathogens. After careful consideration, we feel that it has merit but does not fully meet PLOS Pathogens's publication criteria as it currently stands. Therefore, we invite you to submit a revised version of the manuscript that addresses the points raised during the review process.

Please submit your revised manuscript by September 30. If you will need more time than this to complete your revisions, please reply to this message or contact the journal office at plospathogens@plos.org. When you're ready to submit your revision, log on to https://www.editorialmanager.com/ppathogens/ and select the 'Submissions Needing Revision' folder to locate your manuscript file.

Please include the following items when submitting your revised manuscript:

* A letter that responds to each point raised by the editor and reviewer(s). You should upload this letter as a separate file labeled 'Response to Reviewers'. This file does not need to include responses to any formatting updates and technical items listed in the 'Journal Requirements' section below.

* A marked-up copy of your manuscript that highlights changes made to the original version. You should upload this as a separate file labeled 'Revised Manuscript with Track Changes'.

* An unmarked version of your revised paper without tracked changes. You should upload this as a separate file labeled 'Manuscript'.

We look forward to receiving your revised manuscript.

Kind regards,

Margarita Saiz, Ph.D.

Academic Editor

PLOS Pathogens

Sonja Best

Section Editor

Editor-in-Chief

PLOS Pathogens

orcid.org/0000-0003-2946-9497

Michael Malim

Editor-in-Chief

PLOS Pathogens

orcid.org/0000-0002-7699-2064

**Additional Editor Comments (if provided):**

Dear Dr Li,

Your manuscript has been revised by three experts in the field. Please find their comments at the end of this message. They all agree on the interest and relevance of the work. However, a significant number of concerns have been raised that need to be addressed. The draft should be deeply revised for clarity and correction according to their comments. I´ll be willing to consider a revised manuscript for publication in PLOS Pathogens.

**Journal Requirements:**

If the reviewer comments include a recommendation to cite specific previously published works, please review and evaluate these publications to determine whether they are relevant and should be cited. There is no requirement to cite these works unless the editor has indicated otherwise.

1) Thank you for stating "The cryo-EM density maps and structures for FMDV-OHN-pO18-29 and FMDV-O18-pO18-29 have been deposited into the Electron Microscopy Data Bank (EMDB) and the Protein Data Bank (PDB) with the following accession numbers: FMDV-OHN-pO18-29, EMD-69003, PDB 23IT, and FMDV-O18-pO18-29, EMD-69004, PDB 23IU." However, we were unable to access the accession number using the information provided. Please review and update the Data Availability Statement to ensure that the underlying data are publicly accessible and that all accession numbers, links, or repository details are correct and functional.

2) We have amended your Competing Interest statement to comply with journal style. We kindly ask that you double check the statement and let us know if anything is incorrect.

**Reviewers' Comments:**

Reviewer's Responses to Questions

**Part I - Summary**

Please use this section to discuss strengths/weaknesses of study, novelty/significance, general execution and scholarship.

Reviewer #1: The manuscript presents a well-executed study on antigenic drift in FMDV topotype variants using porcine-derived single B cell neutralizing antibodies. The study is methodologically solid, combining reverse genetics, scFv production, and cryo-electron microscopy to characterize antibody–virus interactions. The work is relevant for understanding antigenic variability and its implications for immune escape and vaccine design in swine populations. Overall, the results are convincing, and the main novelty lies in the functional and structural validation of antigenic determinants in infectious virus systems.

Reviewer #2: Li et al., have used a panel of seven porcine-derived neutralizing monoclonal antibodies (pnAbs) to identify antigenic regions responsible for neutralization escape from porcine immune sera in recent FMDV O/Cathay variants. A charge modification at +2 residue (Q→H/R) downstream the conserved RGD receptor-binding motif on VP1 (within a region well characterized as a relevant B cell antigenic site), modulates sensitivity to different pnAbs and vaccine-induced sera. In addition, cryo-EM structures of one of the pnAbs (pO18-29) in complex with the ancient O/HN/CHA/93 strain (the strain used as a reference in this study) and the recent variant O/18074 revealed a novel conformation-dependent, VP3-centered antigen determinant. Substitutions leading to charge modifications at positions 73 and 134 on VP3 disrupt hydrophobic contacts, hydrogen bonds, and salt bridges, leading the virus to escape different antibodies. Interestingly, these two VP3 residues act cooperatively to regulate antibody recognition.

The authors conclude that the results contribute to elucidate the structural basis of neutralizing evasion in O/Cathay and support the rational design of next-generation broad-spectrum FMDV vaccine against emerging O/Cathay variants.

General comments

The topic of this manuscript is interesting as O/Cathay topotype has accumulated mutations over time associated with antigenic drift that can become a real problem in terms of vaccine effectiveness in different Asian regions. In general, this manuscript is well written and describes a comprehensive and, in my opinion, well conducted original work. Porcine-derived neutralizing mAbs are also interesting and suited tools for the analysis of the antigenic structures recognized by pig sera.

Nevertheless, there are issues regarding data and manuscript presentation as well as discussion that have to be addressed. In this line, the interesting statement at the end of the Abstract “Together, our findings elucidate ….. and provide a molecular framework for rational design of next-generation broad-spectrum FMDV vaccine …… “deserves further attention. The authors can discuss in more detail how these kinds of results can be translated into vaccine optimization and matching, as well as their potential impact on conventional as well as subunit vaccines.

In addition, a comparison of the genetic distances among the type O viruses analyzed, such as a P1 phylogenetic tree, will be valuable to determine correlations between genetic drift (nucleotide substitutions) and the antigenic changes among the O/Cathay variants. The inclusion of such comparison will improve the analyses of the data presented.

Reviewer #3: It has been reported that newly emerging FMDV O/Cathay strains circulating in China can escape neutralization by sera from vaccinated pigs due to the accumulation of mutations that reduce antibody-mediated neutralization and compromise vaccine efficacy. In this manuscript, the authors investigate recently emerged O/Cathay variants that exhibit this immune escape phenotype. The main strength and novelty of the study lies in the structural characterization of the molecular determinants underlying antigenic divergence through the combined use of cryo-electron microscopy and porcine neutralizing monoclonal antibodies.

The authors identify two key antigenic regions involved in immune escape: one located adjacent to the RGD motif in VP1 and a second, previously unrecognized, region in VP3. They demonstrate that two point mutations affecting VP3 disrupt critical molecular interactions and act cooperatively to confer resistance to antibody-mediated neutralization.

The methodology used is appropriate for the study’s objectives and is described in sufficient detail. However, the use of unpaired t-tests with Holm–Šidák correction is not statistically appropriate for this experimental design, which compares vaccine-induced neutralizing antibody titers against the homologous virus and four viral variants. A more appropriate approach would be a one-way ANOVA followed by Dunnett’s multiple-comparison test if the objective is to compare each viral variant exclusively with the homologous virus, or Tukey’s multiple-comparison test if all pairwise comparisons among variants are intended.

Recent outbreaks of foot-and-mouth disease in China have also been caused by two additional serotype O topotypes, Middle East–South Asia (ME-SA) and Southeast Asia (SEA). Is it known whether these topotypes, like Cathay, also exhibit escape from vaccine-induced neutralization? If so, are these escape phenotypes associated with the same mutations and structural alterations identified in the present study? Otherwise, to what extent can the conclusions of this manuscript be generalized to other serotype O topotypes?

Overall, the study is scientifically relevant and provides valuable structural insights into the mechanisms by which emerging FMDV variants evade vaccine-induced immune responses. These findings improve our understanding of the antigenic evolution of the O/Cathay topotype and provide a rational framework for the design of next-generation vaccines with broader cross-protective efficacy against antigenically divergent strains.

**********

**Part II – Major Issues: Key Experiments Required for Acceptance**

Please use this section to detail the key new experiments or modifications of existing experiments that should be absolutely required to validate study conclusions.

Generally, there should be no more than 3 such required experiments or major modifications for a "Major Revision" recommendation. If more than 3 experiments are necessary to validate the study conclusions, then you are encouraged to recommend "Reject".

Reviewer #1: There are no major experimental issues that would be required to support the conclusions of the study. The work is overall well designed and the experimental approaches are appropriate for the objectives of the manuscript.

Reviewer #2: The presence of the mutations selected in the neutralization-scape mutants (Table 1), as well as those in the site-directed mutants (Fig. S1A), should be confirmed in the viral stocks from which the different analyses were conducted.

Reviewer #3: (No Response)

**********

**Part III – Minor Issues: Editorial and Data Presentation Modifications**

Please use this section for editorial suggestions as well as relatively minor modifications of existing data that would enhance clarity.

Reviewer #1: Only minor improvements are suggested to further improve clarity. In particular, the resolution and labeling of some figures could be enhanced for better readability. In addition, a minor error in the abstract regarding antibody naming should be corrected,

These are minor points and do not affect the overall validity or strength of the study.

Reviewer #2: Specific Comments

The type O viruses used in this study in neutralization assays, as well as those employed in the vaccines for pig immunization (as in Fig. 1), should be clearly indicated. In particular, the date of isolation of O/18074 isolate should be provided. As this information is relevant for the data discussion, I recommend its inclusion as a new Table.

The number of passages given to the viral populations, and the stability of the mutations selected in the neutralization-scape mutants (Table 1), as well as those of the site-directed mutants (Fig. S1A) should be clearly indicated and discussed.

Likewise, the rationale for selecting the pnmAb used for Cryo-EM analyses (pO18-29) should be discussed.

Line 131. (Fig. 2B). Why the results with O/18074 in IFA are not shown?

Line 133. The reason why the BLI kinetic analyses revealed that all pmAbs bound O/HN/CHA/93 with greater affinity than the recent variant O/18074 is not evident from what is commented in this section of the Results. This should be revised and the viruses used for the immunization of the pigs for the production of FMDV-specific porcine B-cell repertoires indicated (this reviewer has not been able to access reference 40).

Other points

Abstract, line 22: include “that” in the sentence “Herein, we observed (that) the new emerging …. … “

Abstract, line 32: delete “that” in the sentence: “Moreover, residues 73 and 134 that synergistically regulate pnAbs (pO18-2, pO18-18, pO18-29 and pO18-29) recognition”.

Reviewer #3: Figures 1A–D would benefit from using the same y-axis scale (up to 4 log), which would facilitate direct comparison across panels.

Lines 124–125; Figures 2C–E. The manuscript states that the monoclonal antibodies (mAbs) were derived from the previously established O/18074-specific porcine B-cell receptor repertoire (“55 mAb clones from the previously established O/18074-specific porcine BCR repertoire”). How do the authors explain that several mAbs exhibit higher binding affinity for the ancestral virus O/HN/CHA/93 than for the homologous virus O/18074 (Figures 2C–E)? In this context, Figure 2B should also include the binding reactivity of the mAbs against O/18074, rather than only against the ancestral virus. Alternatively, could there be an error in the manuscript, with the mAbs actually being generated from O/HN/CHA/93?

Figure 5. The figure would be clearer and facilitate comparison if the expanded views of the antibody–antigen interfaces were reorganized into two columns: one for O/HN/CHA/93 and the other for O/18074. Within each column, the panels should be arranged in the order E–F loop, B–C loop, and BB-knob. This layout would allow direct side-by-side comparison of the corresponding structural elements in both viruses. Using the current panel nomenclature, the proposed order would be: A, E, C, G, B, F, D, and H.

Line 259. The statement “All recombinant viruses were successfully rescued (Fig. S2A–B)” appears to cite the wrong figure, as Figure S2 corresponds to the cryo-EM analysis rather than the rescued viruses. The correct citation should be Figures S1A–C.

Figure S3. The y-axis label should be revised from “Dilution (lg)” to “Serum neutralization titers (log).”

**********

PLOS authors have the option to publish the peer review history of their article (what does this mean?). If published, this will include your full peer review and any attached files.

If you choose “no”, your identity will remain anonymous but your review may still be made public.

**Do you want your identity to be public for this peer review?**  For information about this choice, including consent withdrawal, please see our Privacy Policy.

Reviewer #1: No

Reviewer #2: No

Reviewer #3: No

[NOTE: If reviewer comments were submitted as an attachment file, they will be attached to this email and accessible via the submission site. Please log into your account, locate the manuscript record, and check for the action link "View Attachments". If this link does not appear, there are no attachment files.]

**Figure resubmission:**

**Reproducibility:**

?>

# Author comment: Author response to Decision Letter 1

**DOI:** 10.1371/journal.ppat.1014650.r002

8 Sep 2026

Attachment Submitted filename: Response to Reviews-0908.docx

# Peer review: Decision Letter 1

**DOI:** 10.1371/journal.ppat.1014650.r003

**Authors:** Sonja M. Best, Margarita Saiz

22 Sep 2026

Dear Dr Li,

We are pleased to inform that your manuscript, "Structural basis of antigenic drift in FMDV O/Cathay topotype variants revealed by porcine-derived single B-cell neutralizing antibodies", has been editorially accepted for publication at PLOS Pathogens.

Before your manuscript can be formally accepted and sent to production, you will need to complete our formatting changes, which you will receive by email within a week. Please note that your manuscript will not be scheduled for publication until you have made the required changes.

IMPORTANT NOTES

(1) Copyediting and Proofreading: The corresponding author will receive a typeset proof for review, to ensure errors have not been introduced during production. Please review the PDF proof of your manuscript carefully, as this is the last chance to correct any errors. Please note that major changes, or those which affect the scientific understanding of the work, will likely cause delays to the publication date of your manuscript.

(2) Appropriate Figure Files: Please remove all name and figure # text from your figure files. Please also take this time to check that your figures are of high resolution, which will improve the readbility of your figures and help expedite your manuscript's publication. Please note that figures must have been originally created at 300dpi or higher. Do not manually increase the resolution of your files. For instructions on how to properly obtain high quality images, please review our Figure Guidelines, with examples at: http://journals.plos.org/plospathogens/s/figures.

We encourage you to use PACE (http://pace.apexcovantage.com/), a figure-checking tool which helps authors ensure all figures meet PLOS requirements so that figures can publish in the highest quality. PACE is a digital diagnostic and conversion tool for figure files. It will provide information about any failed check(s) and, if able, will automatically convert the figure into an acceptable file that passes quality checks.

To use PACE, you must first register as a user. Then, login and navigate to the UPLOAD tab, where you will find detailed instructions on how to use the tool.

(3) Striking Image: Please upload a striking still image to accompany your article if one is available (you can include a new image or an existing one from within your manuscript). Should your paper be accepted, this image will be considered for our monthly issue image and may also appear on our website to feature your article. Please upload this as a separate file, selecting "striking image" as the file type upon upload. Please also include a separate "Other" file with a caption, including credits and any potential copyright information. Please do not include the caption in the main article file. If your image is from someone other than yourself, please ensure that the artist has read and agreed to the terms and conditions of the Creative Commons Attribution License at http://journals.plos.org/plospathogens/s/content-license. Please note that PLOS cannot publish copyrighted images.

(4) Press Release or Related Media: If you or your institution will be preparing press materials for this manuscript, please inform our press team in advance at plospathogens@plos.org as soon as possible. We ask that you contact us within one week to plan ahead of our fast Production schedule. If you need to know your paper's publication date for related media purposes, you must coordinate with our press team, and your manuscript will remain under a strict press embargo until the publication date and time.

(5) Update your Profile Information: Now that your manuscript has been provisionally accepted, please log into Editorial Manager and update your profile, if needed. Go to http://www.editorialmanager.com/ppathogens, log in, and click on the "Update My Information" link at the top of the page. Please update your user information to ensure an efficient production and billing process.

(6) LaTeX users only: Our staff will ask you to upload a TEX file in addition to the PDF before the paper can be sent to typesetting, so please carefully review our Latex Guidelines http://journals.plos.org/plospathogens/s/latex in the meantime.

Thank you again for supporting Open Access publishing; we are looking forward to publishing your work in PLOS Pathogens.

Best regards,

Margarita Saiz, Ph.D.

Academic Editor

PLOS Pathogens

Sonja Best

Section Editor

PLOS Pathogens

Dominique Soldati-Favre

Editor-in-Chief

PLOS Pathogens

***********************************************************

Reviewer's Responses to Questions

**Part I - Summary**

Please use this section to discuss strengths/weaknesses of study, novelty/significance, general execution and scholarship.<br/><br/>

Reviewer #2: The points and comments raised by this reviewer have been adequately addressed in the revised manuscript.

Reviewer #3: The authors have satisfactorily addressed all comments raised in the first round of review and have incorporated the requested revisions. I have no further comments, and I recommend acceptance of the manuscript for publication.

--------------------

**Part II – Major Issues: Key Experiments Required for Acceptance**

Please use this section to detail the key new experiments or modifications of existing experiments that should be absolutely required to validate study conclusions.<br/><br/>Generally, there should be no more than 3 such required experiments or major modifications for a "Major Revision" recommendation. If more than 3 experiments are necessary to validate the study conclusions, then you are encouraged to recommend "Reject".<br/><br/>

Reviewer #2: The new experimental data requested is now presented and discussed.

Reviewer #3: (No Response)

--------------------

**Part III – Minor Issues: Editorial and Data Presentation Modifications**

Please use this section for editorial suggestions as well as relatively minor modifications of existing data that would enhance clarity.<br/><br/>

Reviewer #2: The minor issues have been addressed.

Reviewer #3: (No Response)

--------------------

PLOS authors have the option to publish the peer review history of their article (what does this mean?). If published, this will include your full peer review and any attached files.

If you choose “no”, your identity will remain anonymous but your review may still be made public.

**Do you want your identity to be public for this peer review?**  For information about this choice, including consent withdrawal, please see our Privacy Policy.

Reviewer #2: No

Reviewer #3: Yes: Esther Blanco

# Editor report: Acceptance letter

**DOI:** 10.1371/journal.ppat.1014650.r004

**Authors:** Sonja M. Best, Margarita Saiz

Dear Dr Li,

We are delighted to inform you that your manuscript, "Structural basis of antigenic drift in FMDV O/Cathay topotype variants revealed by porcine-derived single B-cell neutralizing antibodies," has been formally accepted for publication in PLOS Pathogens.

We have now passed your article onto the PLOS Production Department who will complete the rest of the pre-publication process. All authors will receive a confirmation email upon publication.

Thank you again for supporting open-access publishing; we are looking forward to publishing your work in PLOS Pathogens.

Best regards,

Dominique Soldati-Favre

Editor-in-Chief

PLOS Pathogens
